# Interactions between abundant fungal species influence the fungal community assemblage on limestone

**DOI:** 10.1371/journal.pone.0188443

**Published:** 2017-12-06

**Authors:** Alejandro Morón-Ríos, Sergio Gómez-Cornelio, Benjamin Otto Ortega-Morales, Susana De la Rosa-García, Laila Pamela Partida-Martínez, Patricia Quintana, José Armando Alayón-Gamboa, Silvia Cappello-García, Santiago González-Gómez

**Affiliations:** 1 El Colegio de la Frontera Sur, Ciudad Industrial Lerma, Campeche, México; 2 Departamento de Ingeniería en Biotecnología, Universidad Politécnica del Centro, Carretera Federal Villahermosa-Teapa, Tumbulushal, Tabasco, México; 3 Departamento de Microbiología Ambiental y Biotecnología, Universidad Autónoma de Campeche, Campeche, México; 4 División Académica de Ciencias Biológicas, Universidad Juárez Autónoma de Tabasco, Carr. Villahermosa-Cárdenas, Villahermosa, México; 5 Departamento de Ingeniería Genética, Centro de Investigación y de Estudios Avanzados—Unidad Irapuato, Irapuato, México; 6 Departamento de Física Aplicada, CINVESTAV-IPN, Cordemex, Mérida, México; Universita degli Studi di Pisa, ITALY

## Abstract

The assembly of fungal communities on stone materials is mainly influenced by the differential bioreceptivity of such materials and environmental conditions. However, little is known about the role of fungal interactions in the colonization and establishment of fungal species. We analyzed the effects of intra- and interspecific interactions between 11 species of fungi in oligotrophic and copiotrophic media and on limestone coupons. In a previous study, these species were the most frequently isolated in the epilithic biofilms of limestone walls exposed to a subtropical climate. In the culture media, we found a greater frequency of intra- and interspecific inhibitory effects in the oligotrophic medium than in the copiotrophic medium. On the limestone coupons, all fungi were able to establish; however, the colonization success rate varied significantly. *Cladosporium cladosporioides* had a less extensive colonization in isolation (control) than in dual interactions (coexistence) with other species. *Phoma eupyrena* exhibited the highest colonization success rate and competitive dominance among all tested species. X-ray diffraction (XRD) and scanning electron microscope (SEM) analyses revealed that *Pestalotiopsis maculans* and *Paraconiothyrium* sp. produced calcium oxalate crystals during their growth on coupon surfaces, both in isolation and in dual interactions. Our results demonstrate that interactions between abundant fungal species influence the fungal colonization on substrates, the biomineralization and the fungal community assemblage growing in limestone biofilms.

## Introduction

Studies on the fungal communities that colonize lithic substrates have revealed a diverse assemblage of species [1–3], whose colonization and growth are influenced by different biotic and abiotic factors, including the time of exposure of substrates to the environment, climatic and microclimatic variables, nutrient availability, substrate characteristics and the migration of fungal propagules [4–6]. Interactions among fungal species are also believed to play an important role in epilithic diversity because of the corresponding starting effects of the establishment of certain species on the subsequent colonization of substrates by other species [7, 8]. Most studies on fungal interactions have been conducted with species from wood, soil and aquatic substrates, such as leaf litter and submerged wood [9–12], wherein competitive and facilitation interactions have been found to potentially influence community composition and organization [13, 14].

The types of interactions among fungal species, as well as the responses of particular fungal species to such interactions, depend on the availability of nutrients in the fungal substrate and on environmental conditions [15, 16]. For this reason, from an ecological perspective, the components and nature of a substrate in addition to environmental conditions should be taken into account during the study of fungal interactions and community assemblages. In this respect, fungal interactions can be examined in culture media and on substrates where fungi naturally grow. Several previous studies have used taxonomic and/or molecular methods to study fungal communities and have reported assemblages on lithic substrates that are constituted of hundreds of fungal individuals [2, 4, 17], even though only one out of every four species presented high abundance in their respective communities [2, 18]. The coexistence of fungal species on a substrate implies that they have reached an equilibrium between survival and reproduction. Meanwhile, the low occurrence of a given species in a fungal community could be attributed to non-ideal environmental conditions [19] or competition with other fungi.

Interspecific fungal interactions can have various effects on substrates. In some cases, interspecific interactions enable the optimal degradation of wood substrates [20]. On stone, community interactions likely contribute to biomineralization and, therefore, the formation of soil [6]. To the best of our knowledge, interactions among fungal species on lithic substrates have not yet been studied. We wondered if the interactions among the fungal species inhabiting a given space at a given time are important determinants of the fungal community assemblages in limestone biofilms [21].

Based on a culture-dependent approach, we previously identified the fungal species that were frequently isolated from biofilms adhered to fragments of limestone used to construct buildings, considering the distinct exposure times of these limestone blocks to a subtropical environment in Campeche, México [8]. We observed that the abundance of fungal species in the community depended on the time of exposure of substrates to the environment. This screening also showed that melanized fungi seemed to be more frequent in biofilms. From this assemblage, we selected 11 abundant species with high isolation frequencies to fulfill the objectives of the present study. In particular, our goals were to describe and to evaluate the possible responses of fungi to paired fungal interactions in oligotrophic and copiotrophic culture media in order to infer the possible responses of fungi to such interactions on natural substrates. In addition, we explored the mechanisms of establishment and colonization of fungi in dual interactions on the surface of limestone coupons. On these coupons, we also identified the species that produced calcium oxalate crystals in isolation (controls) and whether this production varied when these species interacted with other species. According to previous studies on fungal community composition on rocks, we hypothesized that abundant fungi and/or fungi with melanized structures in epilithic biofilms have higher competitive dominance in comparison to species with lower abundance on limestone coupons surfaces. In summary, the main objective of the present study was to determine the influence of interactions between abundant fungal species on the colonization and establishment of fungal species and on the fungal community assemblage of limestone.

## Materials and methods

### Selection and identification of fungal species

The fungal community assemblage considered in this study was previously analyzed in Gómez-Cornelio et al. [8], in which 844 individuals corresponding to 202 species were obtained from biofilm samples on limestone walls with different exposure times [1 year (young biofilm), 5 years (middle-aged biofilm), and 10 years (old biofilm)] to a subtropical climate in Campeche, Mexico. These fungi were isolated by washing and the particle filtration technique. This technique reduces the isolation of propagules from spores and favors the isolation of fungi attached to rock particles that likely have an active function on limestone surfaces. From the previously analyzed fungal community [8], we selected 11 species that were isolated a minimum of seven times and that were distinguishable by their phenotypical characteristics, which included their morphological, growth and reproductive structures (Table 1).

**Table 1:** Fungal isolates used for interaction assays in culture media and on limestone coupons in addition to the number of times fungi were isolated in young (Y), middle-aged (M) and old (O) biofilms with distinct environmental exposure times [8].

Species	GenBank accession	Number of isolates from biofilms			Reported lithotypes
		Y	M	O	
Cladosporium cladosporioides*	KX610321	22	10	8	Marble [1, 22, 23], Sandstone [18, 23, 26], Granite [23], Limestone [1, 4, 8], Andesite [23, 24], Mortars [25]
*Curvularia clavata*	KX610320	18	3	1	Limestone [4, 8]
Curvularia lunata*	KX610322	10	105	26	Marble [23], Sandstone [23], Andesite [23]; Limestone [4, 8]
*Fusarium oxysporum*	KX610330	8	40	4	Sandstone [26]; Limestone [4, 8]
Fusarium redolens*	KX610323	14	41	9	Limestone [4, 8]
Hyphomycete sp.	KX610326	-	-	11	Limestone [8]
Myrothecium roridum*	KX610325		12	16	Limestone [8]
*Paraconiothyrium* sp.*	KX610324	19	3	-	Limestone [8]
Pestalotiopsis maculans*	KX610327	5	3	10	Mortars [25], Limestone [4, 27]
Phoma eupyrena*	KX610328	23	12	15	Sandstone [18], Marble [22], Limestone [8]
*Scolecobasidium constrictum*	KX610329	3	4	1	Limestone [4, 8]

*Species used in interaction experiments on limestone coupons.

These species were identified by their micro- and macroscopic characteristics in Gómez-Cornelio et al. [8] and in the present study by sequencing their internal transcribed spacer regions (ITS), as described in López-González et al. [28]. The obtained sequences were deposited in GenBank under Accession Numbers KX610320–KX610330 (Table 1).

### Culture media and incubation conditions of growth rate and fungal interaction

Rocky substrates are generally characterized by oligotrophic conditions [6, 29]. Therefore, to assess the fungal growth rate and fungal interactions, we used two different culture media: a copiotrophic medium consisting of malt extract, which is commonly used for isolating fungi from lithic substrates [1, 3], added with calcium carbonate (MEAC), and an oligotrophic medium (CACO) consisting only of calcium carbonate (CaCO_3_), which is the main component of limestone [6]. The MEAC medium contained 1 g of malt extract, 2 g of CaCO_3_ and 20 g of agar per liter, and the CACO medium contained 2 g of CaCO_3_ and 20 g of agar per liter. The pH of both media was adjusted to 7.7, similar to the pH of limestone. To determine the radial growth rate and the fungal interactions, the culture media assays were incubated at 28°C and 81% relative humidity. These conditions reflect the annual means for temperature and humidity over the last 10 years in the region where the assayed fungi were isolated. These climate data were provided by the Mexican National Water Commission (Comisión Nacional del Agua).

### Paired interactions in culture media

Before analyzing the fungal interactions, we assessed the kinetic growth rate of the 11 evaluated species in quintuplicate. For each assay and each species, we inoculated a 5-mm disc of actively growing mycelium into both culture media, placing each disc at the center of a 90-mm Petri dish without agar. Fungal development and macroscopic morphological characteristics were inspected daily. The measurement of colony growth was stopped when the edges of the Petri dish were reached by the mycelium or, in the case of slow-growing fungi, 17 days after inoculation. The fungal growth rate was classified as slow (0–1 mm d^-1^), moderate (1–3 mm d^-1^) or fast (3–6 mm d^-1^) [30].

Dual interactions (inter- and intraspecific) of the 11 fungal species were then examined, and all possible combinations of fungi were tested in the CACO and MEAC media. The effects of fungal interactions on fungal growth, as well as the response of each fungal species, were evaluated given the different nutritional composition of each culture media. Similar to the growth rate assessment, mycelium discs of 5-mm diameter were taken from an actively growing colony of each fungus and were inoculated in dual assays in both culture media. The two discs were placed 35 mm apart in the same 90-mm Petri dish without agar. To avoid the spore dispersal of fungi with abundant sporulation, we placed the discs in a 20% glycerol solution for 5 minutes. Each assay was performed in quintuplicate. To create controls, we also inoculated all evaluated species in isolation in both culture media. Moderate- and slow-growing fungi were inoculated one and two weeks, respectively, before fast-growing fungi, so fungi with similar growth rates were inoculated within the same time frame [31].

#### Assessment of dual interactions in culture media

We analyzed the results of the assays after 21 days or when one of the species reached the edge of the Petri dish. In particular, we observed the behavior of the fungi in the zone of interaction. We also assessed the antagonism index (AI), a qualitative measure defined as the ability of a species to dominate and to compete with other species. To calculate this index, one of the categories listed in Table 2 was assigned to each interaction; the resulting numerical scores of all assays were then added to achieve a final score per species.

**Table 2:** Types of paired fungal interactions or reactions and corresponding values for abundant fungal species on limestone biofilms. Modified from Yuen et al. [31].

Categories	Interaction/Reaction	Score
A	Mutual intermingling of both species	0
B_1_	Response species overgrows challenge species, growth of challenge species is reduced	1
B_2_	Response species grows up to, on and around challenge species	1
C	Colonies of both species grow until nearly coming into contact and then growth ceases	2
D	Mutual inhibition at a distance between both species	3
E_1_	Challenge species overgrows response species, growth of response species is reduced	4
E_2_	Challenge species grows up to, on and around response species	4

The antagonism index was calculated as follows:

AI = A(n x 1) + B_1_(n x 1) + B_2_(n x 1) + C(n x 2) + D(n x 3) + E_1_(n x 4) + E_2_(n x 4)

where n = number of times that a fungus corresponded with a given category or categories (A, B_1_…E_2_), resulting in the corresponding score; greater value was given to the categories representing inhibitory responses.

In addition to the AI, we assessed the fungal interactions using two other quantitative and complementary measures, the first of which was the percentage of inhibition. For all interactions, we measured the rate of radial growth of each species along a line drawn from the center of each inoculation disc toward the challenging species in each culture medium. We then compared the growth and the macro- and microscopic characteristics of all species with respect to their controls. The overall ability of a species to inhibit the radial growth of competitors was determined through adding its observed percentages of inhibition with respect to all competitors in all assays and comparing the resulting percentage to its growth in the control [28]. The percentages of inhibition were calculated as follows:

% Inhibition = (growth of a given species in its respective control–growth of a given species in presence of an inhibitory species) x 100/growth of a given species in its respective control.

The second quantitative measure was the percentage of resistance; this percentage represented the overall ability of a given species to grow and to resist the presence of other fungi. It was determined by adding the growth percentages of a given species in all dual assays with other fungal species and comparing the resulting percentage to its growth in isolation [30]. The resistance percentages were determined as follows:

% Resistance = 1,100%, which is the sum total inhibition of all species,–Σ([growth of a given fungal species in its respective control dish–growth of the given species when interacting with another species] x 100/growth of a species in its respective control dish).

### Dual interactions on limestone coupons

The assays were performed with seven of the 11 species used in the previous section to differentiate and to identify the typical morphological growth characteristics of these fungi on limestone substrates (Table 1). Inoculations were performed with conidia to promote the colonization of coupon surfaces. The species Hyphomycete sp. and *Scolecobasidium constrictum* were excluded because of their weak sporulation. The species of the genera *Curvularia* and *Fusarium*, which had the largest number of isolates in Gómez-Cornelio et al. [8], were selected for this portion of the study (Table 1). The limestone used as a substrate was quarried in the Yucatan Peninsula, Mexico, and is extracted at a depth of 0.3–2.5 m; this rock is frequently used in the construction and restoration of buildings. This limestone lithotype was hard with low porosity (~5%) and contained shades of red, which indicated the presence of goethite traces. This limestone was also characterized by a high occurrence of pellets and the presence of foraminifera, undetermined bivalves and echinoderm remains embedded in a sparitic matrix [32]. The coupons were cut 2 × 1 × 0.5 cm and then sterilized three times at 121°C for 60 min to reduce the microbial burden [4].

**Fig 1:**
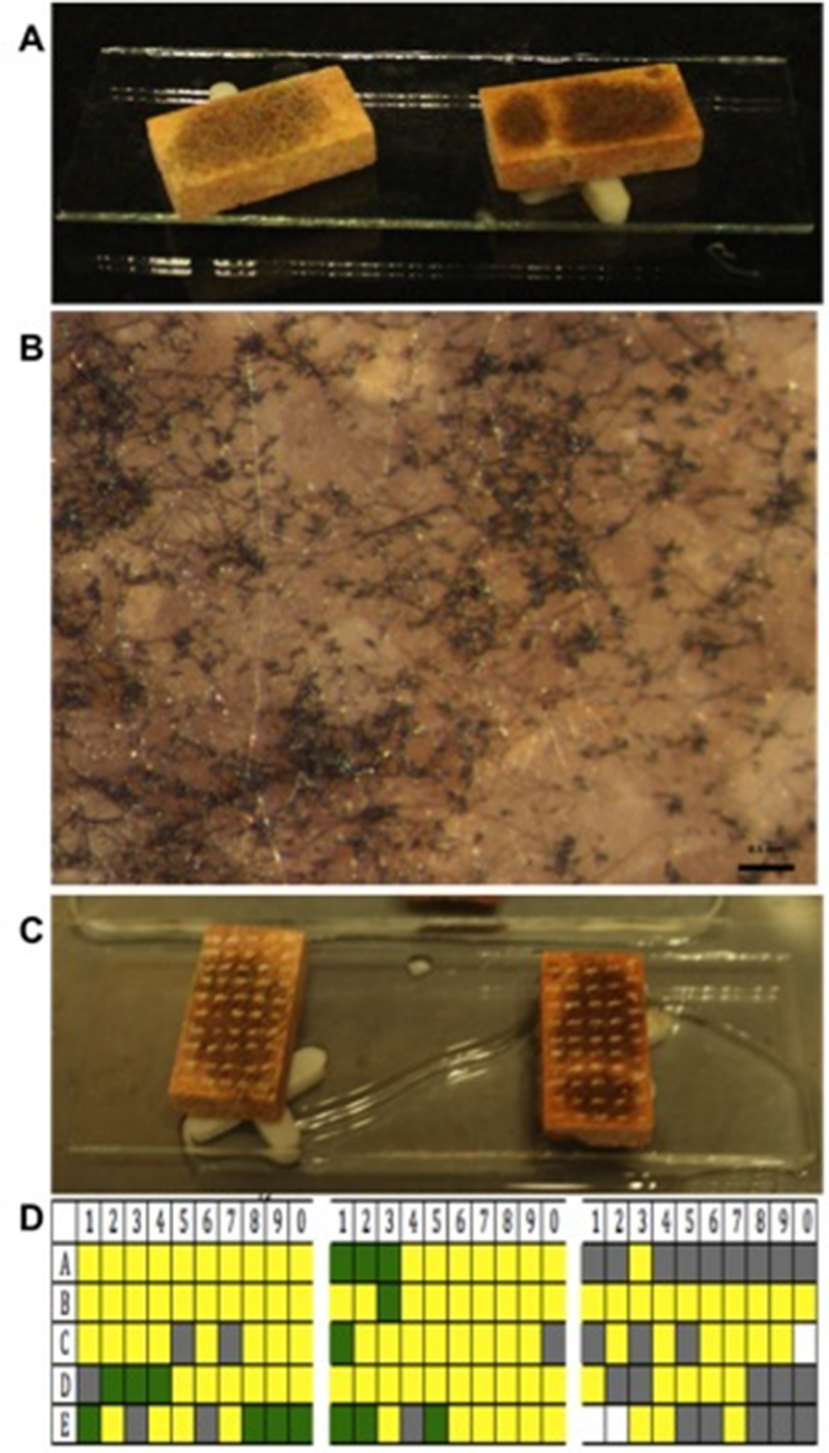
Interaction between *Curvularia lunata* and *Phoma eupyrena*. A) Coupons four months after inoculation, B) microphotography of a coupon, C) surface after sampling by points, D) area colonized by *C*. *lunata* (green), *P*. *eupyrena* (grey) and both species (yellow) and area without growth (white) in triplicate limestone coupons in the CACO medium.

The fungi that were inoculated on the limestone coupons were previously grown in CACO medium. First, the fungal growth on plates was flooded with sterile saline solution (0.85%) containing 0.2% Tween 80 (v/v). Then, for the dual interactions assays on the limestone coupons, the conidia concentrations were adjusted to 6 × 10^4^ conidia mL^-1^; both concentrations to be evaluated were mixed so that the final inoculum suspension per coupon contained 1.2 × 10^5^ conidia mL^-1^. To minimize any bias resulting from density-dependent effects between the dual interactions assays and the controls, the conidia concentration of each species was also adjusted to 1.2 × 10^5^ conidia mL^-1^ per coupon for the control treatments. The conidial suspensions were homogeneously inoculated on coupon surfaces in quadruplicate. We also prepared fungi-free coupons. After inoculation, coupons were placed in square Petri dishes of 10 × 10 cm, which were incubated for four months and inspected every 15 days. We added sterile water to the Petri dishes to maintain moisture constant. The coupons were placed on sterile plastic supports, as shown in Fig 1A, whose function was to prevent coupons from having direct contact with water.

#### Assessment of dual interactions on limestone coupons

After four months, we photographed the coupons inoculated with fungi and analyzed the establishment and morphology of all fungal species in comparison to their controls. The dual interactions on the surface of coupons were examined with a stereomicroscope (Carl Zeiss, Jena, Germany) (Fig 1B). Then, we applied the mycelium re-isolation technique and transferred fungal species to Petri dishes with culture medium to confirm species’ identities [12, 33]. For this, we sectioned the surface of each coupon, including the controls and the coupons with dual interactions, into 50 quadrants of approximately 20 x 20 mm that spanned the entire surface of each coupon (Fig 1C and 1D). We picked each quadrant with a sterile dissecting needle up to a depth of 1 mm and transferred the 50 quadrants to five Petri dishes containing CACO medium. The dishes were incubated at 28°C and were inspected daily for 30 days. We identified the colonies that emerged from the inoculation points and compared them to the pure cultures of known fungal species [34]. In addition, the spaces occupied by each fungus on the limestone coupons were calculated as the percentages of colonization. These percentages were calculated based on the number of quadrants in which species A, B or both species (coexistence) emerged [Fig 1D], which was divided by the 50 quadrants and then multiplied by 100. For the control coupons (species inoculated in isolation), the percentage of colonization was calculated in a similar way based on the number of quadrants in which each species emerged.

### SEM and XRD analysis

We used X-ray diffraction (XRD) to analyze the biominerals formed by the fungal species, focusing on the formation of calcium oxalate crystals (whewellite and weddellite). Scanning electron microscopy (SEM) was used to observe fungal adhesion, colonization and distribution on the surface of limestone coupons inoculated with one species (controls) and with two species (dual interactions) after four months. The XRD analysis was performed directly on coupon surfaces of 2 × 2 cm without any previous treatment. For this analysis, a Bruker Advance D8 diffractometer (Karlsrushe, German) was employed at room temperature using Cu-Kalpha radiation (λ = 1.5418 Å) at the θ–θ configuration and a graphite secondary beam monochromator. X-ray diffraction patterns were recorded between 10–50° (2θ), with a step size of 0.02 at 1.0 s per point. The semi-quantitative phase composition analysis was based on the highest peak intensity (according to the Powder Diffraction File [PDF]) for each crystalline phase registered in the diffractogram, whose sum was required to reach 100%. Therefore, the following equation was applied: % Intensity of F1 = 100(I_F1_/I_F1_ + I_F2_ + I_F3_ … I_Fn_), where I_F1_ + I_F2_ + I_F3_ … I_Fn_ is the intensity of each phase.

The analysis and characterization of the colonization and establishment of fungi on the coupon surfaces were performed using an environmental scanning electron microscope (ESEM, Philips XL30, Eindhoven, The Netherlands). In addition, fragments of coupons were deposited on standard carbon tape for observation, and images of these were taken under low-vacuum conditions (secondary electrons imaging). Finally, the elemental analysis of the crystals formed on the coupon surfaces was carried out via field emission scanning electron microscopy (FESEM, Model JSM-7600F, Jeol Ltd., Tokyo, Japan).

### Statistical analysis

The interaction responses of fungi on culture media and limestone coupons were subjected to analyses of variance (ANOVA) given the established classification criterion. The means of treatments were compared with Tukey's multiple comparison tests [16]. The Levene's test and the Shapiro-Wilk test were used to assess the homogeneity of the variance and normality, respectively, of the data. Statistical analyses were performed in MiniTab 16 (Minitab Inc., State College, PA).

## Results

The fungal species involved in this study were previously identified in a fungal community assemblage on limestone in a subtropical environment of Campeche, Mexico [8]. In particular, we examined the 11 species that were most frequently isolated; some of these species were previously confirmed to colonize diverse rocky substrates and to often coexist with one another under distinct environmental conditions (Table 1). These 11 fungal species were identified using morphological and molecular identification techniques. We identified nine fungi at the species level and one at genus level. Hyphomycete sp. could only be identified at the class level because of its asexual reproductive structures; molecular sequencing and BLAST showed this fungus to be 94% similar to an uncultured fungus.

### Dual interactions in Petri dishes

**Fig 2:**
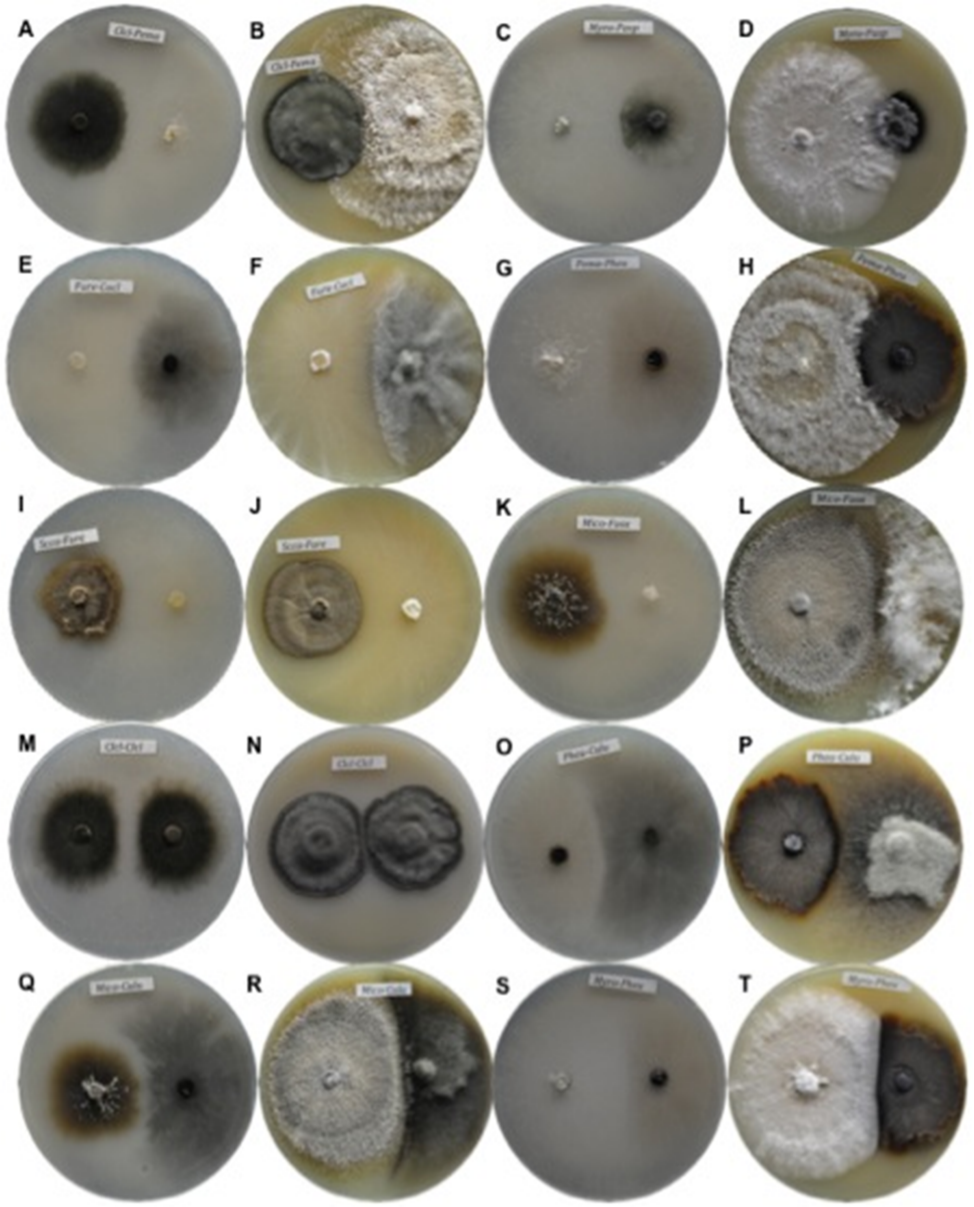
Intra- and interspecific interactions between paired fungi in culture media. Columns 1 and 3: CACO medium; columns 2 and 4: MEAC medium. A and B) *C*. *cladosporioides*–*P*. *maculans*, C and D) *M*. *roridum*–Hyphomycete sp., E and F) *F*. *redolens*–*C*. *clavata*, G and H) *P*. *maculans*–*P*. *eupyrena*, I and J) *S*. *constrictum*–*F*. *redolens*, K and L) *Paraconiotrhyrium* sp.–*F*. *oxysporum*, M and N) *C*. *cladosporioides*–*C*. *cladosporioides*, O and P) *P*. *eupyrena*–*C*. *lunata*, Q and R) *Paraconiothyrium* sp.–*C*. *lunata*, S and T) *M*. *roridum*–*P*. *eupyrena*.

The growth of the evaluated fungi in oligotrophic and copiotrophic culture media showed a wide range of interaction types (Table 2) that were dependent on the species with which they interacted (Fig 2 and S1 Table) and on the culture medium (Table 3 and S2 Table).

**Table 3:** Fungal interaction types, antagonism index (AI) scores and inhibition and resistance percentages per species with respect to all paired species (ten) and itself in MEAC and CACO media. The AI categories are listed in Table 2.

Species	Media	Categories							AI	Inhibition	Resistance
		A	B_1_	B_2_	C	D	E_1_	E_2_			
*Cladosporium cladosporioides*	MEAC	1	4	3	3	-	-	-	19	177.9	994.3
	CACO	4	-	1	2	4	-	-	45	179.5	911.4
*Curvularia clavata*	MEAC	1	1	-	5	-	-	4	85	370.7	496.2
	CACO	-	-	2	2	7	-	-	73	339.9	463.2
*Curvularia lunata*	MEAC	-	-	-	8	1	-	2	73	354.9	480.0
	CACO	-	-	-	3	8	-	-	84	358.3	507.4
*Fusarium oxysporum*	MEAC	1	-	1	7	-	2	-	61	322.3	670.4
	CACO	1	-	-	6	1	-	3	81	417.2	652.2
*Fusarium redolens*	MEAC	-	-	-	2	-	4	5	152	480.9	766.6
	CACO	-	-	-	-	4	1	6	148	422.6	592.6
Hyphomycete sp.	MEAC	1	2	4	4	-	-	-	22	151.0	1027.6
	CACO	2	1	4	2	2	-	-	31	221.0	1097.2
*Myrothecium roridum*	MEAC	-	-	1	5	-	4	1	101	542.1	852.7
	CACO	2	-	1	5	-	2	1	69	485.2	878.1
*Paraconiothyrium* sp.	MEAC	1	-	-	7	-	-	3	76	557.6	721.3
	CACO	-	-	2	4	5	-	-	63	363.1	913.3
*Pestalotiopsis maculans*	MEAC	2	2	-	4	-	2	1	66	347.6	664.2
	CACO	1	2	-	7	-	-	1	46	341.9	636.9
*Phoma eupyrena*	MEAC	1	1	3	3	2	-	1	50	206.3	737.8
	CACO	-	-	-	5	5	-	1	81	297.4	679.0
*Scolecobasidium constrictum*	MEAC	-	1	6	3	1	-	-	28	234.9	938.7
	CACO	1	-	2	4	4	-	-	54	260.8	1072.4

In the intraspecific interactions in the CACO medium, 91% of fungi displayed competitive interactions corresponding with categories C and D (Table 3 and S1 Table), wherein the radial growth of both inocula stopped before coming into contact, or mutual inhibition occurred at a certain distance. In the MEAC medium, mutual intermingling was observed for *Curvularia clavata*, *Fusarium oxysporum*, Hyphomycete sp. and *Pestalotiopsis maculans*, whereas the rest of the species displayed competitive interactions (Fig 2M and 2N and S1 Table).

Several types of interspecific interactions were also observed. Most frequently, both fungal species grew until almost coming into contact with one another (category C), and then growth stopped. This was the most frequent interaction in both MEAC (41%) and CACO (34%) media (Fig 2L, 2N, 2R and 2T and S1 Table). Hyphal intermingling (category A) was observed in 9% and 3.5% of interactions in the CACO and MEAC media, respectively (Fig 2A, Table 3). In 21% of the interactions in the MEAC medium, one species overgrew the other (B_1_ and E_1_), while 32% of species grew up to and around the challenge species (B_2_ and E_2_). Mutual inhibition at a distance (category D) was observed in 34% of the interactions in the CACO medium yet in only 2.5% in the MEAC medium (Fig 2M, 2O and 2Q and Table 3).

These interactions were reflected in the calculated antagonism index (AI) (Table 3), which enabled a clearer understanding of the competitive capacity of fungal species. Two extreme cases were denoted by the scores calculated for the antagonism index. One case was *C*. *cladosporioides* in the MEAC medium, which obtained a low antagonism index score (19). This species exhibited mutual intermingling (category A) with one species and presented interactions characteristic of type B_1_ with four species and of types B_2_ and C with three species each. At the other extreme, *F*. *redolens* in the MEAC medium presented interactions of types E_1_ and E_2_ with four and five species, respectively, and also presented type-C interactions with two species; this behavior corresponds with its high score (152) on the AI (Tables 2 and 3). The AI calculated for other species such as *P*. *maculans*, *P*. *eupyrena* and *M*. *roridum* varied between the culture media.

With respect to inhibition, *M*. *roridum* and *F*. *redolens* showed the highest inhibition percentages in relation to their challenging species in both culture media (Table 3). Meanwhile, other species such as *Paraconiothyrium* sp. and *F*. *oxysporum* presented different inhibition percentages in the dual assays depending on the medium. The inhibition of species by the growth of *Curvularia lunata*, *C*. *clavata* and *P*. *maculans* was moderate, and *C*. *cladosporioides*, Hyphomycete sp., *P*. *eupyrena* and *S*. *constrictum* showed the lowest inhibition ability (Table 3).

Species whose growth was unaffected by the presence of other fungal species were further evaluated by calculating their percentages of resistance. In both culture media, slow-growing species such as *Cladosporium cladosporioides*, Hyphomycete sp. and *S*. *constrictum* displayed high percentages of resistance; therefore, their growth was little affected by the presence of other fungi. However, *F*. *oxysporum*, *M*. *roridum*, *P*. *maculans* and *P*. *eupyrena* showed intermediate resistance, and *C*. *lunata* and *C*. *clavata* displayed the lowest resistance. The resistance of species such as *Paraconiothyrium* sp. and *F*. *redolens* depended on the interaction medium, similar to their inhibition behavior (Table 3).

In the oligotrophic medium, the growth of Hyphomycete sp. increased significantly (P<0.05) in the presence of *C*. *cladosporioides* and *S*. *constrictum* compared to the control (S2 Table). In most interactions, a decrease in radial growth was observed in the presence of other fungal species, and significant differences were observed relative to the controls. However, several species did not exhibit significant differences in colony growth in the presence of at least five of the challenging species, including *C*. *cladosporioides* and Hyphomycete sp. in the MEAC medium and *S*. *constrictum*, Hyphomycete sp. and *Paraconiothyrium* sp. in the CACO medium. Furthermore, *M*. *roridum* and *Paraconiothyrium* sp. led to a decrease in the radial growth rate of all challenge species and of each other (P<0.05) in both culture media (S2 Table).

### Fungal interactions on limestone coupons

We observed the superficial colonization of fungi on all limestone coupons that were inoculated with one (control) or two species (Tables 4 and 5). In the controls, with the exception of *C*. *cladosporioides*, the colonization of coupons was greater than 70%. Significant differences were observed between *C*. *lunata*, *P*. *maculans* and *P*. *eupyrena* with respect to *F*. *redolens* and *C*. *cladosporioides* (Table 4).

**Table 4:** Percentage of limestone coupon surfaces colonized by fungi (controls) after four months of exposure.

Species	Average colonization %
*Cladosporium cladosporioides*	8.7 ± 2.3^c^
*Curvularia lunata*	99.3 ± 1.2^a^
*Fusarium redolens*	74.7 ± 15^b^
*Myrotecium roridum*	88.0 ± 7.2^ab^
*Paraconiothyrium* sp.	87.3 ± 1.2^ab^
*Pestalotiopsis maculans*	94.0 ± 6.0^a^
*Phoma eupyrena*	98.0 ± 2.0^a^

Means (± SE, n = 3) followed by the same letter (^a, b, c^) do not differ significantly according to Tukey’s post hoc test at P≤ 0.05.

**Table 5:** Percentage of limestone coupon surfaces colonized by interacting fungal species after four months of exposure.

Species A	Species B	Species A	Species B	Colonization by both species	Area without colonization
*C*. *cladosporioides*	*C*. *lunata*	–	47.3 ± 28.0^a^	52.0 ± 29.1^a^	0.7 ± 1.2^a^
*C*. *cladosporioides*	*F*. *redolens*	30.0 ± 7.2^b^	52.0 ± 7.2^a^	6.0 ± 4.0^c^	12.0 ± 2.0^c^
*C*. *cladosporioides*	*M*. *roridum*	10.7 ± 18.5^ab^	34.0 ± 28.2^ab^	54.0 ± 19.7^a^	1.3 ± 2.3^b^
*C*. *cladosporioides*	*Paraconiothyrium* sp.	2.0 ± 2.0^b^	2.7 ± 2.3^b^	–	95.3 ± 1.2^a^
*C*. *cladosporioides*	*P*. *maculans*	0.7 ± 1.2^c^	72.0 ± 8.7^a^	–	27.3 ± 9.5^b^
*C*. *cladosporioides*	*P*. *eupyrena*	–	98.0^a^	1.3 ± 1.2^b^	0.7 ± 1.2^b^
*C*. *lunata*	*F*. *redolens*	90.7 ± 4.2^a^	1.3 ± 1.2^b^	0.7 ± 1.2^b^	7.3 ± 5.0^b^
*C*. *lunata*	*M*. *roridum*	21.3 ± 4.2^b^	2.0 ± 3.5^c^	75.3 ± 4.2^a^	1.3 ± 1.2^c^
*C*. *lunata*	*Paraconiothyrium* sp.	99.3 ± 1.2^a^	–	–	0.7 ± 1.2^b^
*C*. *lunata*	*P*. *maculans*	92.0 ± 6.0^a^	–	3.3 ± 2.3^b^	4.7 ± 6.4^b^
*C*. *lunata*	*P*. *eupyrena*	10.0 ± 8.7^b^	19.3 ± 23.4^b^	68.7 ± 18.1^a^	2.0 ± 3.5^b^
*F*. *redolens*	*M*. *roridum*	48.7 ± 11.4^a^	28.7 ± 7.0^b^	2.7 ± 2.3^c^	20.0 ± 4.0^bc^
*F*. *redolens*	*Paraconiothyrium* sp.	2.7 ± 1.2^b^	2.0 ± 2.0^b^	–	95.3 ± 2.3^a^
*F*. *redolens*	*P*. *maculans*	89.3 ± 3.1^a^	–	1.3 ± 1.2^c^	9.3 ± 3.1^b^
*F*. *redolens*	*P*. *eupyrena*	–	88.7 ± 8.1^a^	0.7 ± 1.2^b^	10.7 ± 9.0^b^
*M*. *roridum*	*Paraconiothyrium* sp.	95.3 ± 4.6^a^	–	0.7 ± 1.2^b^	4.0 ± 5.3^b^
*M*. *roridum*	*P*. *maculans*	44.0 ± 30.2^a^	19.3 ± 33.5^a^	28.0 ± 17.1^a^	8.7 ± 8.3^a^
*M*. *roridum*	*P*. *eupyrena*	39.3 ± 3.1^a^	39.3 ± 5.0^a^	12.7 ± 3.1^b^	8.7 ± 5.0^b^
*Paraconiothyrium* sp.	*P*. *maculans*	6.0 ± 5.3^bc^	82.7 ± 4.2^a^	0.7 ± 1.2^c^	10.7 ± 3.1^b^
*Paraconiothyrium* sp.	*P*. *eupyrena*	–	88.7 ± 12.9^a^	4.7 ± 5.0^b^	6.7 ± 8.1^b^
*P*. *maculans*	*P*. *eupyrena*	–	98.7 ± 1.2^a^	0.7 ± 1.2^b^	0.7 ± 1.2^b^

Means (± SE, n = 3) followed by the same letter (^a, b, c^) do not differ significantly in different fungal interactions according to Tukey’s post hoc test at P≤ 0.05.–: without colonization.

In the dual interactions, *P*. *eupyrena* displayed greater dominance during colonization than *C*. *cladosporioides*, *F*. *redolens*, *Paraconiothyrium* sp. and *P*. *maculans*. In the interaction between *P*. *eupyrena* and *M*. *roridum*, we observed competition for space during colonization: Each fungus colonized 39.3% of the coupon surface, yet the fungi also coexisted across a small portion (12.7%) of the coupon surface. In turn, *C*. *lunata* dominated the limestone surface when interacting with *F*. *redolens*, *Paraconiothyrium* sp. and *P*. *maculans* (P<0.05) and was able to coexist with *C*. *cladosporioides*, *M*. *roridum* and *P*. *eupyrena* in the same space on the stone surface (with a recovery rate of higher than 54%). Furthermore, *F*. *redolens* displayed greater dominance during colonization than *C*. *cladosporioides*, *M*. *roridum* and *P*. *maculans*. However, *P*. *maculans* dominated the stone surface when interacting with *Paraconiothyrium* sp. and *C*. *cladosporioides*, and *M*. *roridum* dominated *Paraconiothyrium* sp. and *P*. *maculans*. Finally, *C*. *cladosporioides* coexisted with *M*. *roridum* across 54% of the coupon surface (Table 5).

As expected, the XRD analysis revealed a high proportion of calcite on all coupon surfaces. The presence of calcium oxalates was observed on coupons colonized by *Paraconiothyrium* sp. and *P*. *maculans* in isolation, which had distinct proportions of whewellite and weddellite. Whewellite production was detected on the coupons of *P*. *maculans* interacting with *M*. *roridum* and *C*. *cladosporioides*. Weddellite production was also detected in the interactions of *P*. *maculans* with *Paraconiothyrium* sp. and *P*. *eupyrena* (Table 6).

**Table 6:** Production of calcium oxalates (whewellite and weddellite) and calcite on limestone coupon surfaces inoculated with fungal species in isolation or paired with other species according to phase XRD analysis.

Fungal interaction	Calcite	Whewellite	Weddellite
*Paraconiothyrium* sp.	73.0%	25.2%	1.8%
*Pestalotiopsis maculans*	97.5%	0.8%	1.7%
*C*. *cladosporioides–P*. *maculans*	97.9%	2.1%	-
*M*. *roridum*–*P*. *maculans*	97.2%	2.8%	-
*Paraconiothyrium* sp.–P. *maculans*	97.8%	-	2.2%
*P*. *maculans*–*P*. *eupyrena*	98.6%	-	1.4%

**Fig 3:**
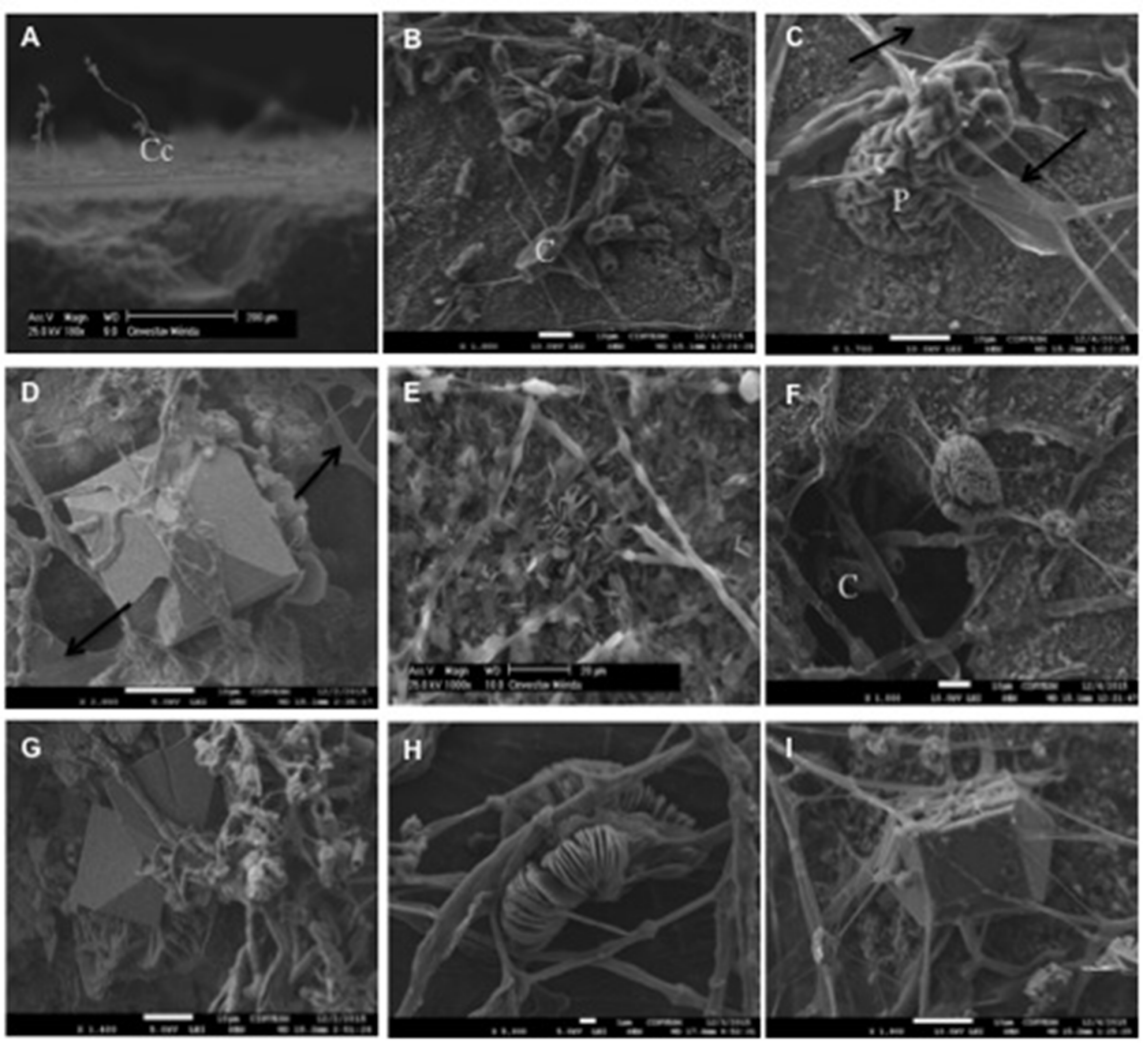
SEM images of limestone coupon surfaces colonized by paired fungi after four months depicting the calcium oxalate crystals produced by some species. A) *C*. *cladosporioides*–*Paraconiothyrium* sp., B) *C*. *lunata*, C) *Paraconiothyrium* sp.–*P*. *eupyrena*, D) *C*. *lunata*–*P*. *maculans*, E) *Paraconiothyrium* sp., F) *M*. *roridum*–*P*. *maculans*, G) *C*. *cladosporioides*–*P*. *maculans*, H) *P*. *maculans*, and I) *P*. *maculans*–*P*. *eupyrena*. Black arrows indicate polymeric fungal production. Cc: Conidiophore; C: conidia; P: pycnidia.

In the SEM analysis, we observed the preference of *C*. *cladosporioides* for colonizing the edge of limestone coupons (Fig 3A), whereas *C*. *lunata* was a successful colonizer across the entire stone surface and produced both conidia and hyphae (Fig 3B). Also, pycnidia formation was seen in the culture of *P*. *eupyrena* (Fig 3C). In most interactions, adhesion to the substrate was enabled by the production of extracellular polymeric material (Fig 3C and 3D, arrows). The different types of crystals produced by the evaluated fungi are listed in Table 6 (Fig 3E–3I). We found calcium oxalate crystals in the interaction between *C*. *lunata* and *P*. *maculans*, although these were not detected in the XRD analysis.

## Discussion

In addition to the diverse biotic and abiotic factors that determine fungal community assembly, our findings also show that interactions between fungal species influence fungal community assembly on limestone. Indeed, as hypothesized, antagonistic responses were frequently observed between species, mainly between those that were differentially abundant in biofilms adhered to limestone at different environmental exposure times (Table 1) [8]. Interactions between fungal species on stone have not been previously documented, except for a mutualistic interaction between a fungus and an alga [7]. Thus, our study shows the first evidence that epilithic fungi interact with neighboring fungi distinctly depending on the culture medium and the identity of neighboring species (Fig 2). These fungal interactions were evaluated by calculating the antagonism index, the inhibition and resistance percentages and the growth of each mycelium toward challenge species in oligotrophic and copiotrophic culture media (Table 3 and S2 Table). The use of two nutritionally dissimilar culture media enabled a better understanding the responses of the interacting species [9, 35]. All intraspecific interactions recorded in the oligotrophic medium and 73% of the interactions observed in the copiotrophic medium led to combative defense responses (categories C and D). The encountered inhibition resulting from intra- and interspecific fungal interactions in the culture media could be due to the production of diffusible metabolites in the agar [36].

In the interspecific interactions, we frequently observed the inhibition of one or both fungal species (Table 3), which could indicate somatic incompatibility [37]. In addition, hyphal intermingling, which may result from cooperative or neutral interactions, was observed between a low proportion of species (Table 3 and S1 Table) [11]. Meanwhile, the growth of Hyphomycete sp. was significantly stimulated during interactions (S2 Table), although this appears to be unusual for soil fungi [9]. In addition, the high frequency of *C*. *lunata*, *F*. *redolens*, *M*. *roridum* and *Paraconiothyrium* sp. in different biofilms on limestone (Table 1) could be explained by their rapid growth rate; these species may inhibit the radial growth of neighboring fungi and exclude slower-growing species [15, 37] as they compete for the few available nutrients and spaces (pores, cracks or edges) on limestone surfaces.

The interactions in the culture media differed from those observed on the limestone coupons. Most fungal species inhibited each other’s growth on agar, yet fungi growing on limestone coupons developed different colonization and competitive dominance strategies. Notably, 90% of the interacting fungi colonized more than 70% of the limestone coupon surfaces (Table 5). This finding suggests that resource partitioning in habitats and species’ abiotic preferences likely define species’ fundamental niches, although fungal interactions and competition among species ultimately determine species’ realized niche [38]. Also, the pore system of rocks could play an important role in determining preferred niches, as some fungi may prefer colonizing the superficial pores of the rocks (Fig 3F), while others extend their hyphae to the rock interior [39].

The limestone coupons were not exposed to natural conditions that would allow fungi to obtain nutrients from external sources such as dust, particulate environmental contaminants, rainwater or phototrophic biomass, nor were nutrients artificially added [25]. In particular, phototrophic organisms are considered to be pioneer species in the establishment of microbial communities on bare rocks [24, 40]. Thus, we can infer that the evaluated fungal species lived at the expense of their metabolic waste via cellular death or exopolymer release on the stone surface (Fig 3C and 3D) [6]. Fungi that produce exopolymeric substances have a competitive advantage over those that do not produce such substances. These substances enable fungi to adhere to limestone and to therefore colonize and establish in biofilms yet can also be used by other species within the community as a source of nutrients [41, 42].

In general, limestone coupons inoculated with a single fungal species, which served as the controls, displayed extensive surface colonization of fungi (Table 3). *Cladosporium cladosporioides* was one exception; this fungus colonized less than 10% of the coupon surfaces and displayed preference for inhabiting the edges of limestone coupons (Fig 3A). This fungus colonized limestone coupons to a larger surface when interacting with *C*. *lunata*, *F*. *redolens*, or *M*. *roridum* (Table 5), while in the CACO medium often presented mutual mycelial intermingling. This suggests that *C*. *cladosporioides* need to grown in association with other species in order to obtain nutrients from mycelium death, metabolic waste or external sources [25]. However, *C*. *cladosporioides* caused inhibition when interacting with some species in culture media (Table 3 and S1 Table), suggesting that this species has the capacity to production of inhibitory compounds [43].

The species *P*. *eupyrena*, *C*. *lunata* and *F*. *redolens* (Table 5) showed higher colonization success when interacting with other species on limestone coupon surfaces. This findings explains the high AI of these species in the oligotrophic medium (CACO) (Table 3) and supports our hypothesis that the species with high abundance in biofilms at different environmental exposure times (Table 1) antagonized the species with lower isolation frequency in our previous study [8]. Additionally, the morphology of certain species, e.g., melanized structures, such as the hyphae and pycnidia of *P*. *eupyrena* and the hyphae and conidiophores of *C*. *lunata*, may serve as an adaptive mechanism that is beneficial to survival and that enables these species to compete under the stressful environmental conditions on limestone [4, 22]. In addition, *F*. *redolens*, which has hyaline structures, appears to prefer colonizing small cracks and pores and likely penetrates rocks, thus excluding other species. On limestone coupons, *M*. *roridum* and *P*. *maculans* presented formation of sporodochia and acervuli, respectively; these structures could facilitate their permanence on rocks and their dominance during colonization and therefore encourage their dispersion over coupon surfaces despite the presence of other species or nutrient deficiencies (Table 5). These findings differ from those of Giannantonio et al. [25], who did not observe acervuli production in *P*. *maculans* on concrete coupons despite having added nutrients. Therefore, we believe that limestone and its oligotrophic conditions may be a fundamental niche for *P*. *maculans*.

Some previous studies on fungal succession and interactions between species on other substrates show that persistent species or fungi in old/late stages are strong competitors, while fungi in young/early stages are weak competitors [31, 33]. In contrast, the findings of other studies [10, 44] and of the present study suggest that frequent species, regardless of their presence in young, middle-aged or old-stage biofilms, are strong competitors on limestone, but especially in young biofilms. In particular, species on young biofilms that can compete for nutrients and that seek initial protection from stressful environment conditions can subsequently colonize the substrate at a higher frequency in comparison to other species of the fungal community assemblage; this initial colonization of species and the resulting formation of biofilms also enables the establishment of other species at later stages. In old-stage biofilms in hostile environments such as limestone, competitive exclusion can decrease as nutrients are added from autotrophic or heterotrophic organisms, leading to an increase in species richness. A similar finding was reported by Gómez-Cornelio et al. [4, 8].

Finally, with respect to the biofilms of different ages (Table 1), *Paraconiothyrium* sp. was abundant in young biofilms and *P*. *maculans* in old biofilms. Both produced monohydrated (whewellite) and dihydrated (weddellite) calcium oxalate (Fig 3F and 3H and Table 6). The replacement of *Paraconiothyrium* sp. by *P*. *maculans* in the old biofilm suggests that these species are functionally redundant in the fungal succession on limestone surfaces. This could also indicate that rock is a transitory substrate of *Paraconiothyrium* sp. (fundamental niche), whereas soil may be its realized niche [45]. However, both fungi were weak competitors when interacting with other species on limestone coupons. In the control coupons, *P*. *maculans* produced both types of calcium oxalate (whewellite and weddellite). However, in some interactions, this fungus produced only one type of calcium oxalate, while no crystals were observed in other interactions (Table 6). This suggests that the coexistence of *P*. *maculans* with other species could result in a specialized and synergic effect that favors the functionality of biofilms on rock substrates [44]. Indeed, our results corroborate that several key fungi were the main biomineralizing agents of rocky substrates and produced calcium oxalates as a result of their metabolism [22, 42]. We only analyzed the surface colonization of compact limestone, yet we do not rule out the possibilities of hyphal penetration and internal colonization of the limestone, as fungi have been previously demonstrated to colonize the interior of rocks [46]. Further studies are needed to understand the additional mechanisms that influence fungal community composition and interactions between community members. In multi-species fungal communities, the frequency and coexistence of species could be a function of species interactions [47]. Hence, evaluating the interactions of multiple species can help to uncover the different mechanisms that are involved in such interactions and to enable a better understanding of how species interactions regulate the roles of fungi on stone substrates. In general, these studies will contribute toward a greater understanding of inhibition mechanisms in community complexes of more than two species.

## Conclusions

Interactions between abundant fungi at different environmental exposure times can influence fungal colonization and community assembly in biofilms on limestone substrates. The fungi analyzed in this study corresponded with those frequently isolated in a previous study. Many of these fungi displayed competitive interactions, mainly in culture media, and exhibited dominance in the surface colonization of limestone coupons. Moreover, several species produced calcium oxalates and may be capable of modulating or regulating biomineralization on limestone surfaces depending on the species with which they interact. These findings have implications for understanding the ecosystem functions of fungi in terms of biodegradation and pedogenesis.

To our knowledge, this is the first study that provides evidence of the interactions between fungal species isolated from lithic substrates and that examines the effect of fungal interactions on the colonization and establishment of fungal species in culture media and stone coupons under laboratory conditions. Notably, we found a high percentage of colonization when frequent fungal species interacted on limestone coupons without the presence of autotroph pioneer organisms or added nutrients. Also, interactions between fungal species could affect fungal successional patterns and also facilitate or inhibit the growth of other species that biomineralize stone surfaces. The effects of species interactions and the resulting conditions could therefore enable or prevent the permanence of certain species on limestone substrates.

## Supporting information

S1 Table Types of interactions/responses between paired fungal species in MEAC (M) and CACO (C) media. Top row = response species; first column = challenge species. The corresponding categories are listed in Table 2. Clcl: *C*. *cladosporioides*, Cucl: *C*. *clavata*, Culu: *C*. *lunata*, Fuox: *F*. *oxysporum*, Fure: *F*. *redolens*, Hyph: Hyphomycete sp., Myro: *M*. *roridum*, Para: *Paraconiothyrium* sp., Pema: *P*. *maculans*, Pheu: *P*. *eupyrena*, Scco: *S*. *constrictum*.
(DOCX)

S2 Table Hyphal growth (cm) of each fungus in isolation (control) and in interaction with fungal pairs. M = MEAC medium; C = CACO medium. Means (± SE, n = 5) followed by the same letter do not differ significantly between the two media according to Tukey’s post hoc test at P ≤ 0.05. Clcl: *C*. *cladosporioides*, Cucl: *C*. *clavata*, Culu: *C*. *lunata*, Fuox: *F*. *oxysporum*, Fure: *F*. *redolens*, Hyph: Hyphomycete sp., Myro: *M*. *roridum*, Para: *Paraconiothyrium* sp., Pema: *P*. *maculans*, Pheu: *P*. *eupyrena*, Scco: *S*. *constrictum*.
(DOCX)

S1 Data Microsoft Excel database of responses in interactions to calculate the antagonism index. (XLSX)

S2 Data Microsoft Excel database to calculate the percentage of inhibition and resistance of fungi in the interactions in culture medium. (XLSX)

S3 Data Microsoft Excel database to determine the inhibition of the growth of each fungal species in the interactions in MEAC and CACO. (XLSX)

S4 Data Microsoft Excel database to calculate the percentage of surface colonization on the calcareous rock coupons. (XLSX)

S5 Data Diffractograms of the XRD analysis after four months that the fungi interacted on the surface of limestone coupons. (ZIP)
