# Supplementary material for: Interactions between abundant fungal species influence the fungal community assemblage on limestone
Source: PLoS One. 2017 Dec 6;12(12):e0188443. doi: 10.1371/journal.pone.0188443 (PMC5718416; doi:10.1371/journal.pone.0188443)
Supplement: S1 Table — Top row = response species; first column = challenge species. The corresponding categories are listed in Table 2. Clcl: C. cladosporioides, Cucl: C. clavata, Culu: C. lunata, Fuox: F. oxysporum, Fure: F. redolens, Hyph: Hyphomycete sp., Myro: M. roridum, Para: Paraconiothyrium sp., Pema: P. maculans, Pheu: P. eupyrena, Scco: S. constrictum. (DOCX) [file pone.0188443.s001.docx]

| **S1 Table. Types of interactions/responses between paired fungal species in MEAC (M) and CACO (C) media.** | | | | | | | | | | | | | | | | | | | | | | |
| --- | --- | --- | --- | --- | --- | --- | --- | --- | --- | --- | --- | --- | --- | --- | --- | --- | --- | --- | --- | --- | --- | --- |
|  | Clcl | | Cucl | | Culu | | Fuox | | Fure | | Hyph | | Myro | | Para | | Pema | | Pheu | | Scco | |
|  | M | C | M | C | M | C | M | C | M | C | M | C | M | C | M | C | M | C | M | C | M | C |
| Clcl | C | D | B_2_ | D | B_2_ | D | B_1_ | A | B_1_ | B_2_ | C | A | B_1_ | A | B_2_ | D | A | A | B_2_ | C | C | C |
| Cucl | E_2_ | D | A | D | C | D | C | C | B_1_ | B_2_ | E_2_ | D | C | B_2_ | C | D | C | C | E_2_ | D | E_2_ | D |
| Culu | E_2_ | D | C | D | C | D | C | D | C | C | C | D | C | C | C | D | C | C | D | D | E_2_ | D |
| Fuox | E_1_ | A | C | C | C | D | A | C | B_2_ | C | C | E_2_ | C | C | C | E_2_ | C | C | C | C | E_1_ | E_2_ |
| Fure | E_1_ | E_2_ | E_1_ | E_2_ | E_1_ | C | E_2_ | C | C | C | E_2_ | E_2_ | E_2_ | E_2_ | C | E_2_ | E_1_ | E_1_ | E_2_ | C | E_2_ | E_2_ |
| Hyph | C | A | B_2_ | D | B_2_ | D | C | B_2_ | B_2_ | B_2_ | A | A | B_1_ | B_1_ | B_2_ | C | B_1_ | B_2_ | C | B_2_ | C | C |
| Myro | E_1_ | A | C | E_2_ | E_1_ | C | C | C | B_2_ | B_2_ | E_1_ | E_1_ | C | C | C | C | E_1_ | E_1_ | C | C | E_2_ | A |
| Para | E_2_ | D | C | D | C | D | C | B_2_ | C | B_2_ | E_2_ | C | C | C | C | D | C | C | A | D | E_2_ | C |
| Pema | A | A | C | C | C | C | C | C | B_1_ | B_1_ | E_1_ | E_2_ | B_1_ | B_1_ | C | C | A | C | E_1_ | C | E_2_ | C |
| Pheu | E_2_ | C | B_2_ | D | B_2_ | D | C | C | B_2_ | C | C | E_2_ | C | C | A | D | B_1_ | C | D | D | D | D |
| Scco | C | C | B_2_ | D | B_2_ | D | B_1_ | B_2_ | B_2_ | B_2_ | C | C | B_2_ | A | B_2_ | C | B_2_ | C | D | D | C | D |
| Top row = response species. First column = challenge species. The corresponding categories are listed in Table 2.  Clcl: *C*. *cladosporioides,* Cucl: *C*. *clavata*, Culu: *C*. *lunata,* Fuox: *F*. *oxysporum*, Fure: *F*. *redolens*, Hyph: Hyphomycete sp., Myro: *M*. *roridum*, Para: *Paraconiothyrium* sp., Pema: *P*. *maculans*, Pheu: *P*. *eupyrena*, Scco: *S*. *constrictum*. | | | | | | | | | | | | | | | | | | | | | | |
