# Supplementary material for: Interactions between abundant fungal species influence the fungal community assemblage on limestone
Source: PLoS One. 2017 Dec 6;12(12):e0188443. doi: 10.1371/journal.pone.0188443 (PMC5718416; doi:10.1371/journal.pone.0188443)
Supplement: S2 Table — M = MEAC medium; C = CACO medium. Means (± SE, n = 5) followed by the same letter do not differ significantly between the two media according to Tukey’s post hoc test at P ≤ 0.05. Clcl: C. cladosporioides, Cucl: C. clavata, Culu: C. lunata, Fuox: F. oxysporum, Fure: F. redolens, Hyph: Hyphomycete sp., Myro: M. roridum, Para: Paraconiothyrium sp., Pema: P. maculans, Pheu: P. eupyrena, Scco: S. constrictum. (DOCX) [file pone.0188443.s002.docx]

| **S2 Table. Hyphal growth (cm) of each fungus in isolation (control) and in interaction with fungal pairs.** | | | | | | | | | | | | |
| --- | --- | --- | --- | --- | --- | --- | --- | --- | --- | --- | --- | --- |
|  | Control | Clcl | Cucl | Culu | Fuox | Fure | Hyph | Myro | Para | Pema | Pheu | Scco |
| Clcl M | 17.4 ± 0.9^ab^ | 13.6 ± 0.9^ef^ | 16.4 ± 0.5^abcd^ | 16.8 ± 0.8^abcd^ | 15.8 ± 0.4^bcde^ | 17.6 ± 2.2 ^ab^ | 15.6 ± 0.5^bcde^ | 13.6 ± 1.1^ef^ | 14.8 ± 0.4^cde^ | 16.6 ± 0.5^abcd^ | 16.6 ± 0.5^abcd^ | 15.6 ± 0.5^bcde^ |
| Clcl C | 18.4 ± 0.9^a^ | 12.3 ± 0.7^f^ | 14.6 ± 1.8^de^ | 17.4 ± 0.5^ab^ | 17.0^abc^ | 14.8 ± 1.3^cde^ | 14.8 ± 0.4^cde^ | 13.6 ± 1.1^ef^ | 15.6 ± 0.9^bcde^ | 16.8 ± 0.8^abcd^ | 17.2 ± 0.8^ab^ | 13.6 ± 0.5^ef^ |
| Cucl M | 53.2 ± 1.3^a^ | 36.8 ± 1.3^bc^ | 17.8 ± 1.5^de^ | 17.2 ± 0.4^de^ | 17.8 ± 1.5^de^ | 15.0 ± 1.9^de^ | 39.2 ± 6.1^b^ | 18.2 ± 1.1^d^ | 12.6 ± 1.8^e^ | 19.8 ± 2.7^d^ | 36.4 ± 3.9^bc^ | 33.2 ± 1.5^c^ |
| Cucl C | 38.6 ± 2.6^b^ | 17.4 ± 0.9^de^ | 16.8 ± 1.3^de^ | 15.4 ± 2.5^de^ | 15.8 ± 1.5^de^ | 16.0 ± 2.2^de^ | 20.2 ± 2.6^d^ | 12.6 ± 2.1^e^ | 15.2 ± 2.3^de^ | 17.4 ± 0.5^de^ | 16.6 ± 0.9^de^ | 15.4 ± 1.9^de^ |
| Culu M | 53.0 ± 3.9^a^ | 26.6 ± 1.1^ef^ | 16.8 ± 0.8^hijklm^ | 18.8 ± 0.8^hijkl^ | 26.0 ± 0.7^efg^ | 13.4 ± 1.1^lm^ | 30.2 ± 5.7^de^ | 14.8 ± 2.4^klm^ | 13.6 ± 3.8^lm^ | 22.6 ± 3.6^fgh^ | 38.4 ± 3.6^bc^ | 33.2 ± 0.8^cd^ |
| Culu C | 43.2 ± 1.3^b^ | 25.6 ± 1.1^efg^ | 22.0 ± 2.0^fghi^ | 17.2 ± 0.8^hijklm^ | 15.6 ± 1.5^jklm^ | 16.0 ± 0.7^ijklm^ | 22.6 ± 4^fgh^ | 11.4 ± 2.6^m^ | 21.2 ± 2.7^fghij^ | 20.2 ± 2.3^ghijk^ | 26.0 ± 4.3^efg^ | 22.0 ± 1.2^fghi^ |
| Fuox M | 27.0 ± 2.9^de^ | 21.6 ± 0.9^efgh^ | 17.0 ± 0.7^ghij^ | 14.0 ± 1.0^hij^ | 17.6 ± 2.3^fghij^ | 11.0 ± 2.2^j^ | 22.8 ± 2.2^defg^ | 13.2 ± 1.9^ij^ | 12.2 ± 3.3^j^ | 16.6 ± 2.4^ghij^ | 18.4 ± 1.7^fghij^ | 16.6 ± 1.5^ghij^ |
| Fuox C | 41.0 ± 8.3^a^ | 37.4 ± 2.6^ab^ | 16.4 ± 0.5^ghij^ | 21.0 ± 2.3^efgh^ | 15.6 ± 0.5^ghij^ | 18.2 ± 1.1^fghij^ | 36.8 ± 5.5^abc^ | 17.4 ± 2.8^fghij^ | 25.0 ± 6.3^def^ | 20.4± 4.8^efghi^ | 29.8 ± 4.0^bcd^ | 29.4 ± 1.9^cd^ |
| Fure M | 59.8 ± 1.1^ab^ | 60.6 ± 0.9^a^ | 36.0 ± 1.4^e^ | 34.0 ± 3.8^ef^ | 46.0 ± 2.7^d^ | 16.0^h^ | 53.6 ± 1.3^c^ | 34.2 ± 4.3^ef^ | 26.4 ± 2.5^g^ | 41.6 ± 3.2^d^ | 55.0^bc^ | 55.0^bc^ |
| Fure C | 51.4 ± 2.4^c^ | 43.0 ± 1.2^d^ | 29.6 ± 0.9^fg^ | 18.0 ± 0.7^h^ | 16.4 ± 1.3^h^ | 17.2 ± 1.1^h^ | 34.0 ± 2.9^ef^ | 20.2 ± 1.5^h^ | 33.2 ± 3.9^ef^ | 32.2 ± 0.8^ef^ | 30.8± 1.8^fg^ | 30.0 ± 1.9^fg^ |
| Hyph M | 15.2 ± 0.8^bcdef^ | 15.0 ± 0.7^bcdef^ | 15.2 ± 0.8^bcdef^ | 16.2 ± 0.8^abcde^ | 15.0 ± 1.9^bcdef^ | 12.6 ± 0.5^efg^ | 17.0 ± 1^abc^ | 8.6 ± 1.9^h^ | 12.0 ± 0.7^fgh^ | 13.0 ± 1.6^defg^ | 15.0 ± 1.6^bcdef^ | 16.6 ± 2.8^abcd^ |
| Hyph C | 13.8 ± 0.8^cdefg^ | 19.0 ± 3.1^a^ | 12.2 ± 0.4^fgh^ | 13.0 ± 1.2^defg^ | 10.2 ± 1.3^gh^ | 12.2 ± 1.1^fgh^ | 15.6 ± 2.5^abcdef^ | 16.6 ± 1.5^abcd^ | 12.0 ± 1.9^fgh^ | 10.4 ± 0.9^gh^ | 12.6 ± 1.5^efg^ | 17.6 ± 1.5^ab^ |
| Myro M | 33.0 ± 1.9^a^ | 28.0 ± 1.9^bcd^ | 27.2 ± 1.6^cde^ | 26.6 ± 3.5^cdef^ | 23.4 ± 0.5^efghi^ | 25.0^cdefg^ | 32.4 ± 1.8^ab^ | 18.2 ± 1.3 ^j^ | 19.2 ± 1.3 ^ij^ | 29.2 ± 2.6^abc^ | 25.4 ± 0.9^cdefg^ | 26.8 ± 4.9^cde^ |
| Myro C | 29.2 ± 0.8^abc^ | 29.4 ± 0.5^abc^ | 24.2 ± 0.8^befgh^ | 23.4 ± 1.1^efghi^ | 22.2 ± 1.3^fghij^ | 21.0 ± 2.1^ghij^ | 23.4 ± 1.5^efghi^ | 18 ± 1.6 ^j^ | 20.2 ± 1.1^hij^ | 25.0 ± 1.6^cdefg^ | 23.2 ± 1.8^efghi^ | 26.4 ± 1.1^cdef^ |
| Para M | 41.4 ± 1.5^a^ | 35.0 ± 0.7^b^ | 27.8 ± 1.3^d^ | 29.0 ± 1.0^cd^ | 31.6 ± 2.9^bc^ | 25.6 ± 0.9^de^ | 23.6 ± 1.3^ef^ | 15.6 ± 1.1^jk^ | 17.8 ± 1.1^ghij^ | 26.8 ± 0.8^de^ | 33.2 ± 1.3^b^ | 32.6± 1.8^b^ |
| Para C | 21.0 ± 1.6^fg^ | 15.8 ± 1.3^ijk^ | 18.8 ± 1.8^ghij^ | 18.8 ± 1.6^ghij^ | 19.2 ± 2^ghi^ | 16.8 ± 0.8^hij^ | 20.0 ± 1.9^gh^ | 13.0 ± 0.7^k^ | 17.0 ± 1.6^hij^ | 17.4 ± 2.3^hij^ | 16.8 ± 0.4^hij^ | 18.2 ± 1.5^ghij^ |
| Pema M | 37.4 ± 1.5^a^ | 32.4 ± 1.9^bc^ | 22.2 ± 3.3^ef^ | 26.0 ± 5.3^de^ | 18.8 ± 2.4^fgh^ | 14.6 ± 0.9^hi^ | 34.4 ± 1.5^ab^ | 10.8 ± 1.5^ij^ | 8.2 ± 1.1^j^ | 17.6 ± 0.5^gh^ | 31.6 ± 1.5^bc^ | 31.8 ± 1.9^bc^ |
| Pema C | 29.8 ± 0.4^cd^ | 17.6 ± 1.5^gh^ | 18.4 ± 1.1^fgh^ | 16.2 ± 0.8^h^ | 16.0 ± 1.4^h^ | 15.4 ± 0.5^h^ | 21.8 ± 0.4^efg^ | 10.4 ± 0.9^ij^ | 17.8 ± 2.6^fgh^ | 16.4 ± 0.5^h^ | 18.2 ± 1.9^fgh^ | 21.6 ± 0.9^efg^ |
| Pheu M | 23.8 ± 1.3^ab^ | 24.4 ± 1.7^ab^ | 14.4 ± 2.3^efgh^ | 14.4 ± 1.1^efgh^ | 18.2 ± 1.8^de^ | 12.6 ± 0.9^hi^ | 19.8 ± 0.4^cd^ | 15.0 ± 3.2^efgh^ | 10.2 ± 0.8^i^ | 18.2 ± 1.6^de^ | 15.4 ± 0.5^efgh^ | 13.0 ± 1.0^ghi^ |
| Pheu C | 27.6 ± 0.9^a^ | 22.4 ± 2.5^bc^ | 14.4 ± 0.5^defg^ | 15.2 ± 1.1^efgh^ | 14.8 ± 0.4^efgh^ | 14.0 ± 0.7^fghi^ | 23.4 ± 2.9^bc^ | 13.2 ± 1.9^ghi^ | 15.6 ± 1.1^efgh^ | 17.2 ± 1.6^def^ | 16.8 ± 0.8^defg^ | 18.2 ± 2.3^de^ |
| Scco M | 18.6 ± 0.5^ab^ | 16.0 ± 1.2^bcdefg^ | 15.0 ± 1.0^cdefg^ | 16.4 ± 1.1^bcde^ | 16.4 ± 1.1^bcde^ | 15.4 ± 0.5^cdefg^ | 19.2 ± 0.8^a^ | 13.4 ± 1.1^gh^ | 13.8 ± 0.8^efgh^ | 15.4 ± 0.5^cdefg^ | 17.0 ± 0.7^abc^ | 16.6 ± 0.9^abcd^ |
| Scco C | 15.2 ± 0.8^cdefg^ | 16.6 ± 0.5^abcd^ | 16.2 ± 2.5^bcdef^ | 16.4 ± 2.4^bcde^ | 14.4 ± 1.1^cdefg^ | 15.0 ± 1.4^cdefg^ | 14.6 ± 1.8^cdefg^ | 11.6 ± 0.9^h^ | 13.6 ± 0.9^fgh^ | 15.2 ± 0.4^cdefg^ | 15.4 ± 1.1^cdefg^ | 14.0 ± 0.7^defgh^ |
| M = MEAC medium; C = CACO medium. Means (± SE, n = 5) followed by the same letter do not differ significantly between the two media according to Tukey’s post hoc test at P≤ 0.05.  Clcl: *C*. *cladosporioides,* Cucl: *C*. *clavata*, Culu: *C*. *lunata,* Fuox: *F*. *oxysporum*, Fure: *F*. *redolens*, Hyph: Hyphomycete sp., Myro: *M*. *roridum*, Para: *Paraconiothyrium* sp., Pema: *P*. *maculans*, Pheu: *P*. *eupyrena*, Scco: *S*. *constrictum*. | | | | | | | | | | | | |
