# Supplementary material for: Interactions between abundant fungal species influence the fungal community assemblage on limestone
Source: PLoS One. 2017 Dec 6;12(12):e0188443. doi: 10.1371/journal.pone.0188443 (PMC5718416; doi:10.1371/journal.pone.0188443)
Supplement: S5 Data — (ZIP) [file pone.0188443.s007.zip › S6 Data/DRX.docx]

**Production of calcium oxalates (whewellite and weddellite) and calcite on limestone coupons surface inoculated with fungal species both in isolation and paired with other species according to phase XRD analysis.**

| **Interaction** | **Calcita** | **Whewellita** | **Weddellita** |
| --- | --- | --- | --- |
| *Paraconiothyrium* sp. | 73% | 25.2% | 1.8% |
| *Pestalotiopsis maculans* | 97.5% | 0.8% | 1.7% |
| Clcl-Pema | 97.9% | 2.1% | 0 |
| Myro-Pema | 97.2% | 2.8% | 0 |
| Pasp-Pema | 97.8% | 0 | 2.2% |
| Pema-Pheu | 98.6% | 0 | 1.4% |
