# Supplementary figures and images for: Interactions between abundant fungal species influence the fungal community assemblage on limestone
Source: PLoS One. 2017 Dec 6;12(12):e0188443. doi: 10.1371/journal.pone.0188443 (PMC5718416; doi:10.1371/journal.pone.0188443)

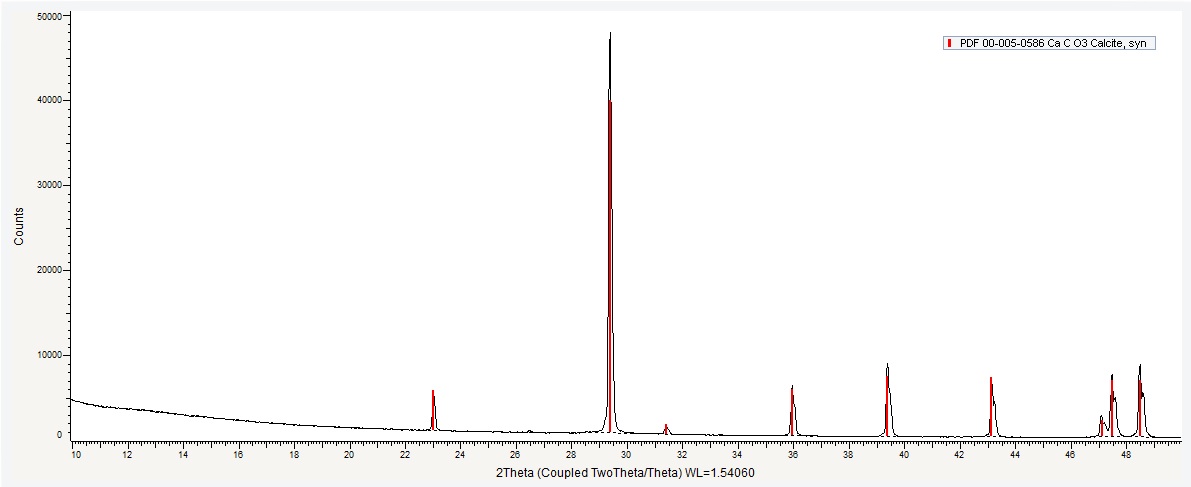

Supplement: S5 Data — (ZIP) [file pone.0188443.s007.zip › S6 Data/CLCL.jpg]

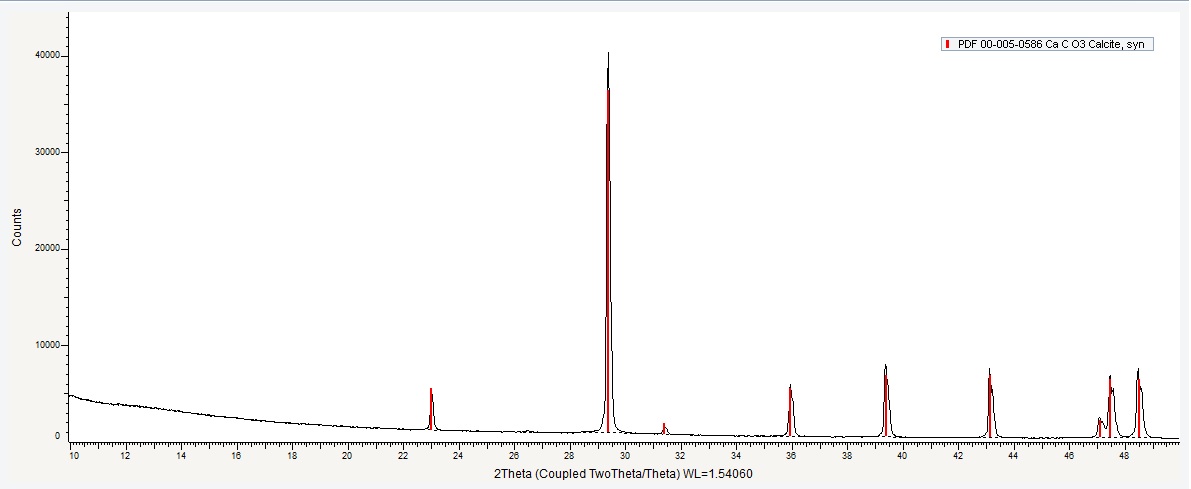

Supplement: S5 Data — (ZIP) [file pone.0188443.s007.zip › S6 Data/CLCLCULU.jpg]

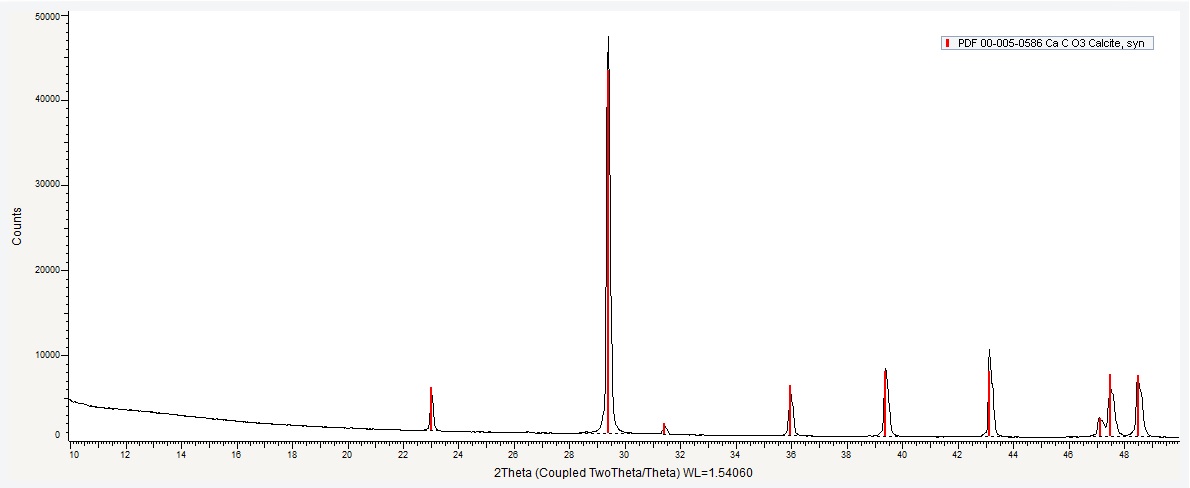

Supplement: S5 Data — (ZIP) [file pone.0188443.s007.zip › S6 Data/CLCLFURE.jpg]

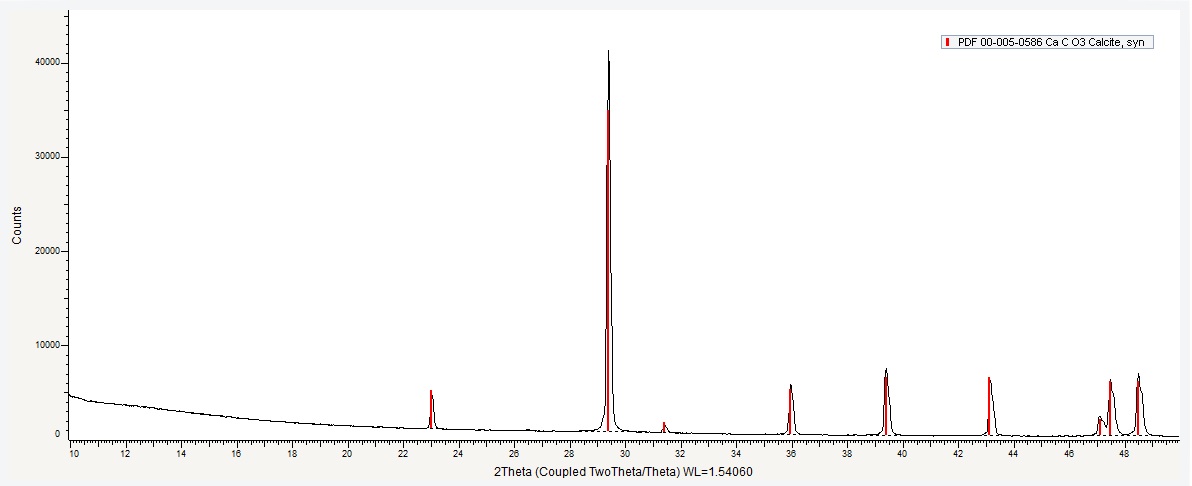

Supplement: S5 Data — (ZIP) [file pone.0188443.s007.zip › S6 Data/CLCLMICO.jpg]

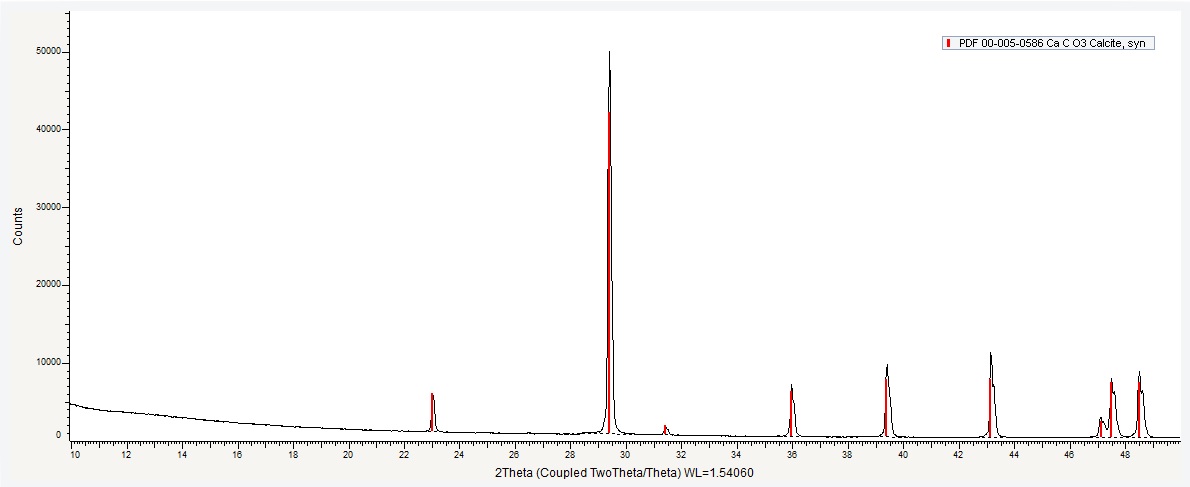

Supplement: S5 Data — (ZIP) [file pone.0188443.s007.zip › S6 Data/CLCLMYRO.jpg]

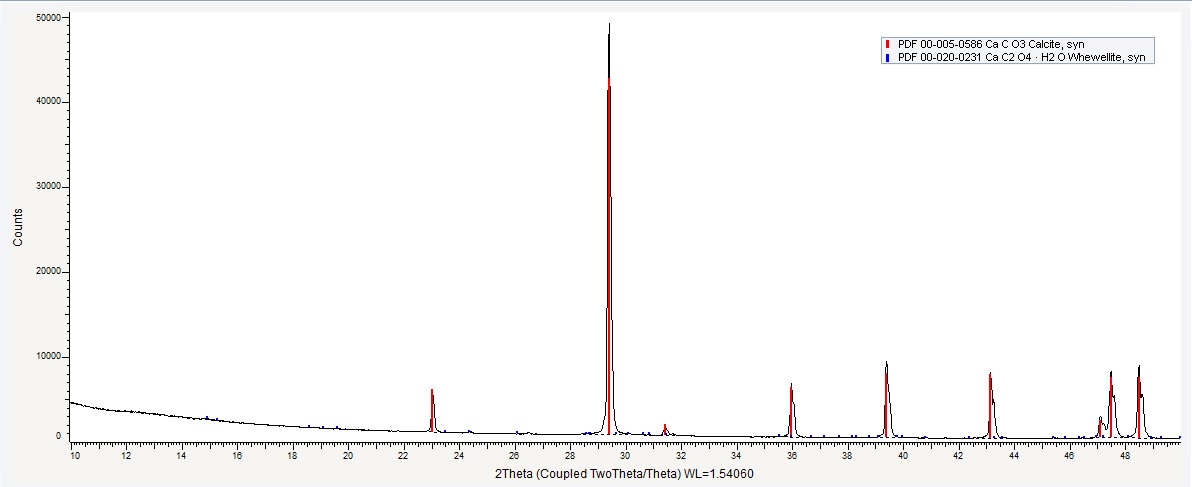

Supplement: S5 Data — (ZIP) [file pone.0188443.s007.zip › S6 Data/CLCLPEMA.jpg]

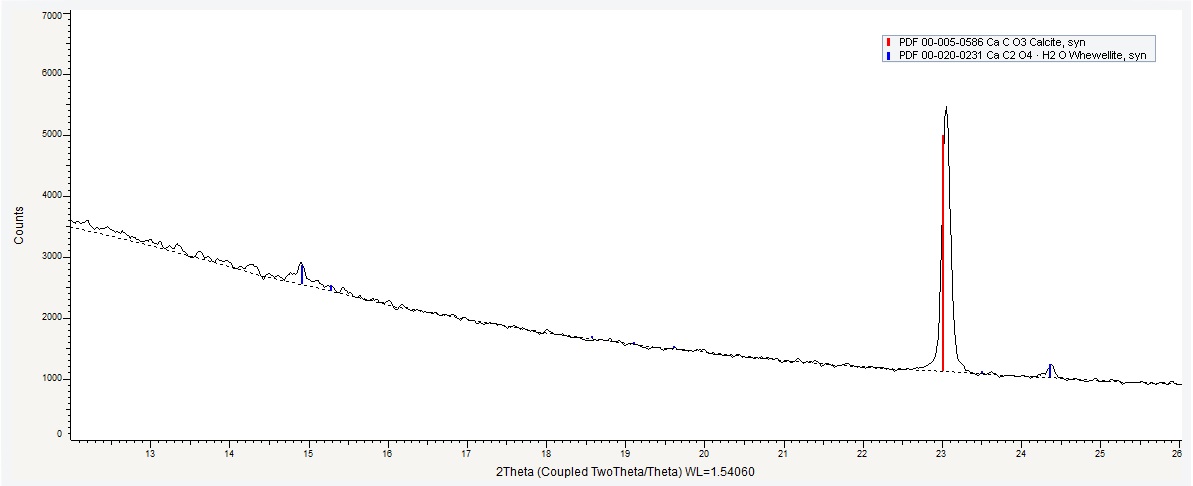

Supplement: S5 Data — (ZIP) [file pone.0188443.s007.zip › S6 Data/CLCLPEMA_2.jpg]

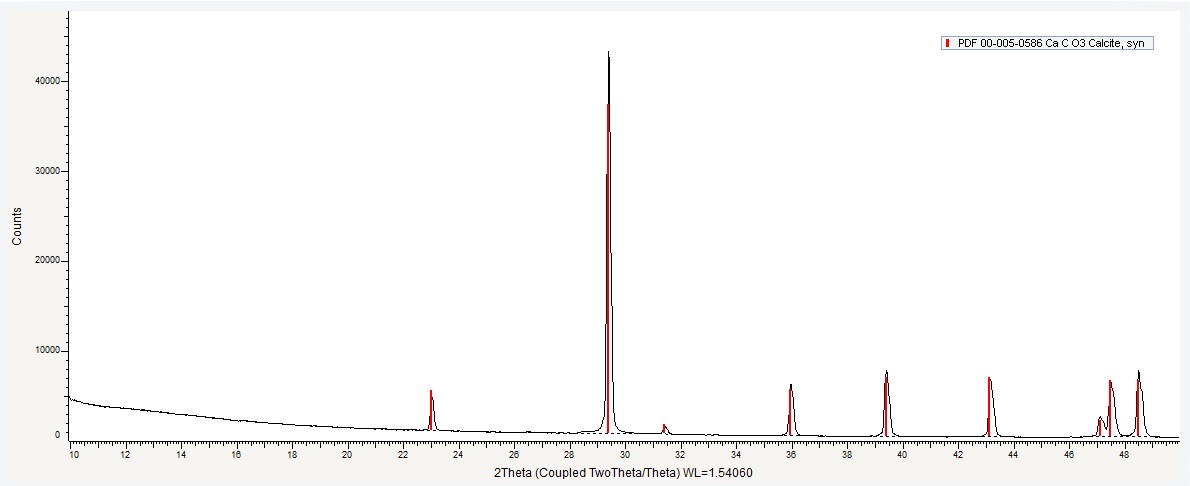

Supplement: S5 Data — (ZIP) [file pone.0188443.s007.zip › S6 Data/CLCLPHEU.jpg]

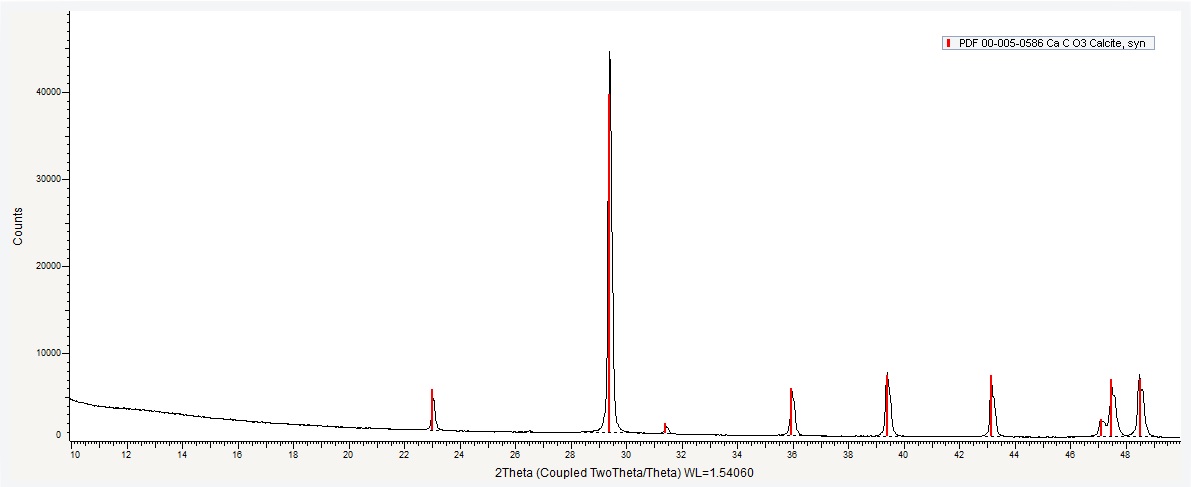

Supplement: S5 Data — (ZIP) [file pone.0188443.s007.zip › S6 Data/CULU.jpg]

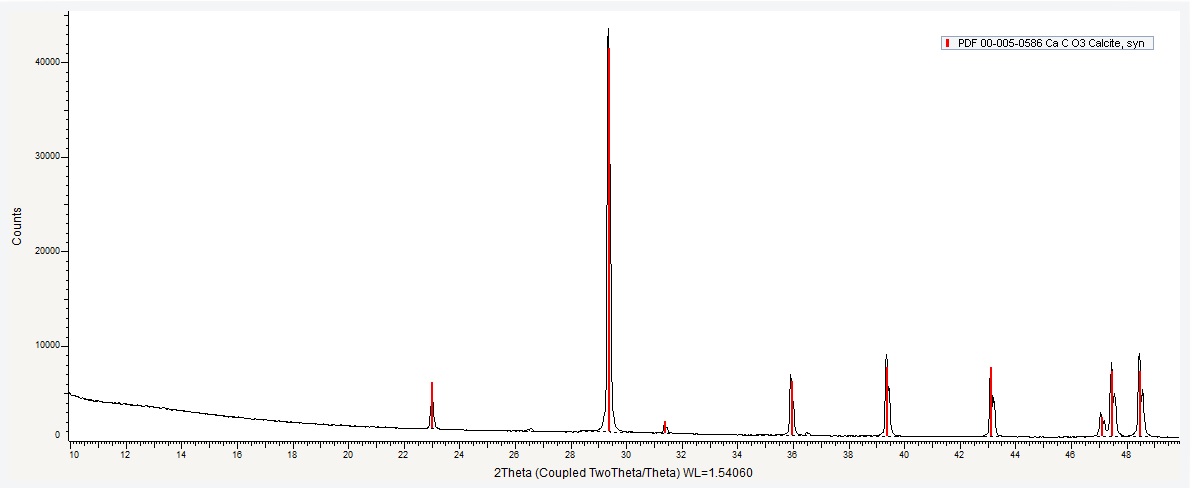

Supplement: S5 Data — (ZIP) [file pone.0188443.s007.zip › S6 Data/CULUMICO.jpg]

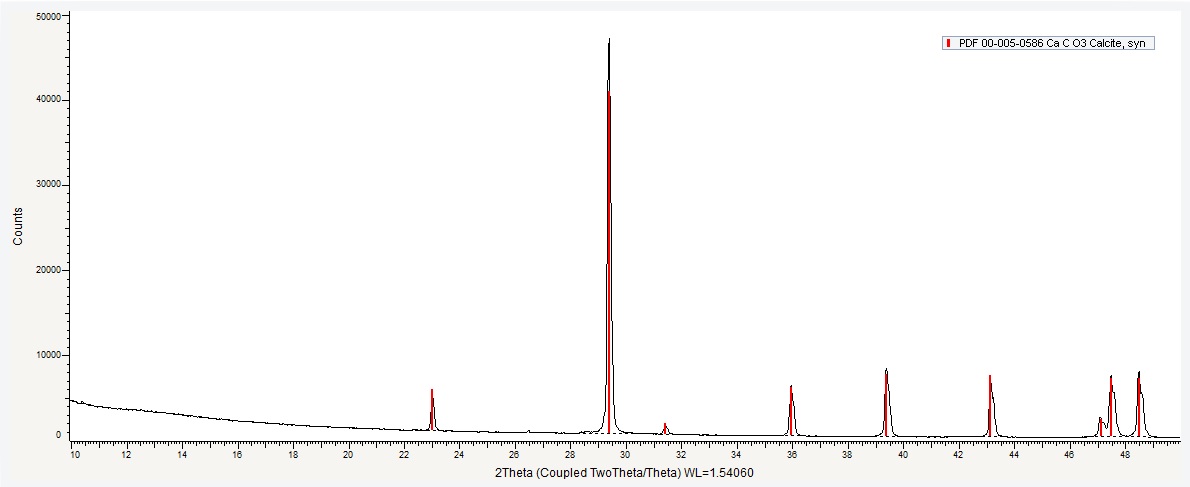

Supplement: S5 Data — (ZIP) [file pone.0188443.s007.zip › S6 Data/CULUMYRO.jpg]

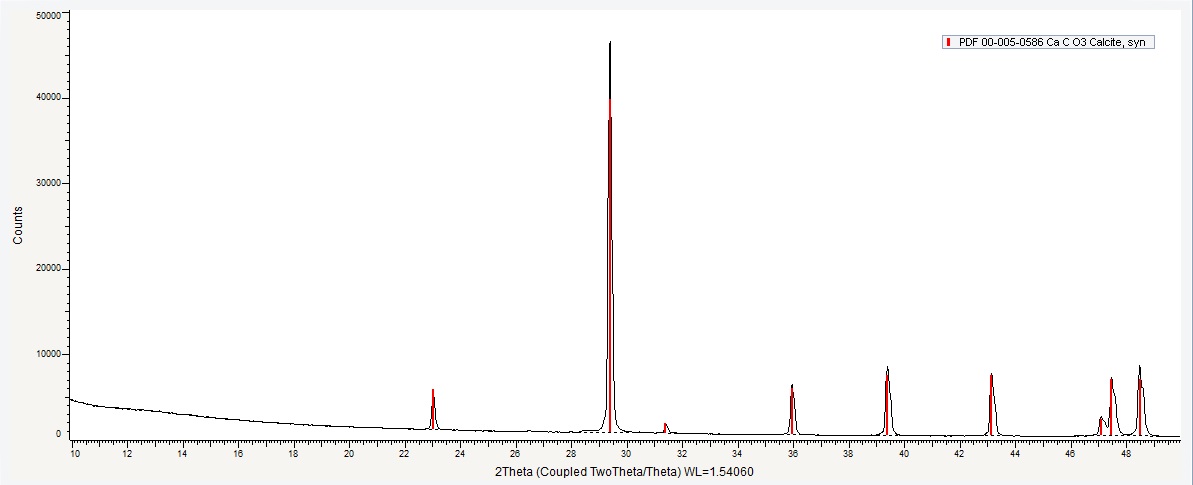

Supplement: S5 Data — (ZIP) [file pone.0188443.s007.zip › S6 Data/CULUPEMA.jpg]

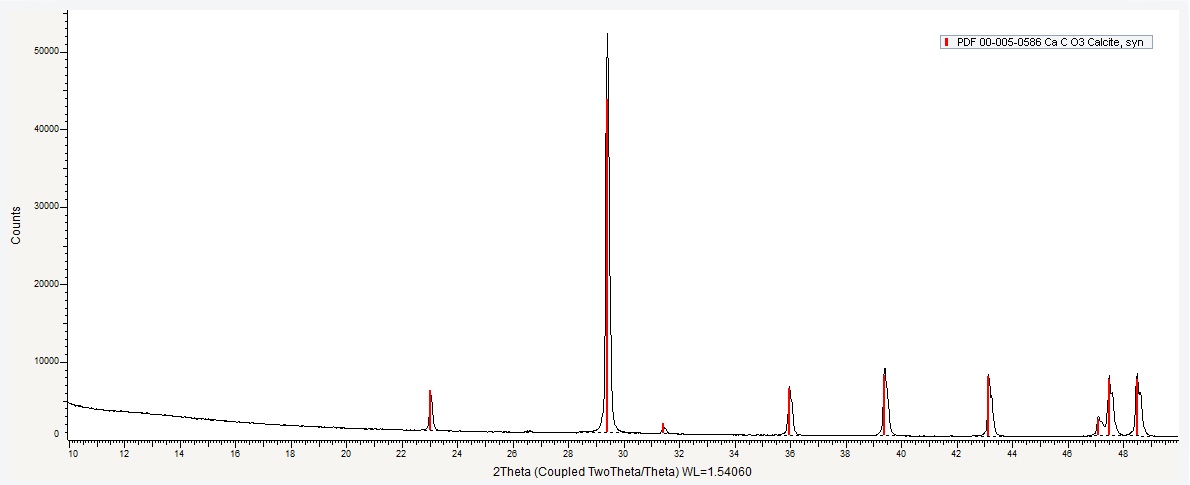

Supplement: S5 Data — (ZIP) [file pone.0188443.s007.zip › S6 Data/CULUPHEU.jpg]

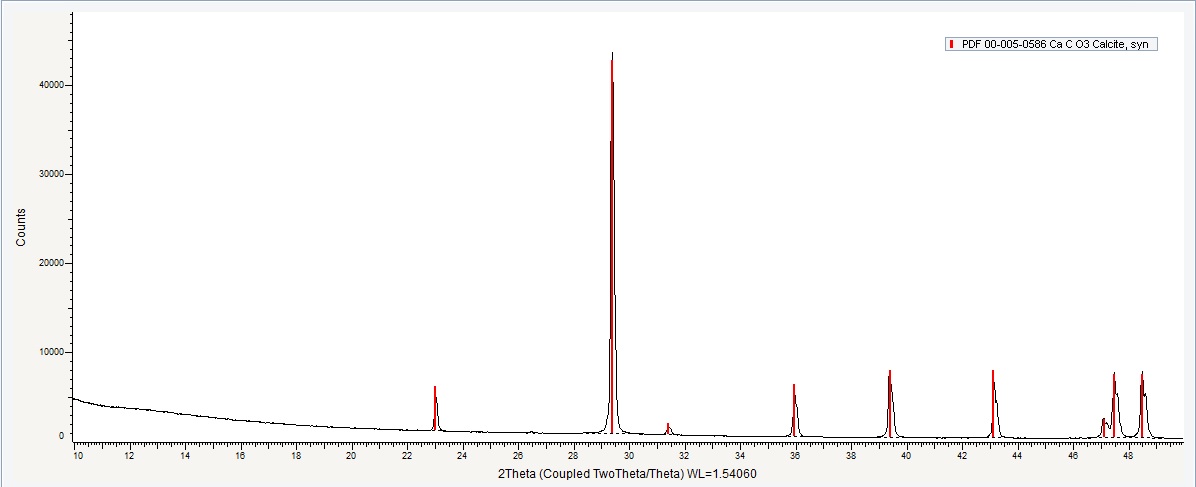

Supplement: S5 Data — (ZIP) [file pone.0188443.s007.zip › S6 Data/FURE.jpg]

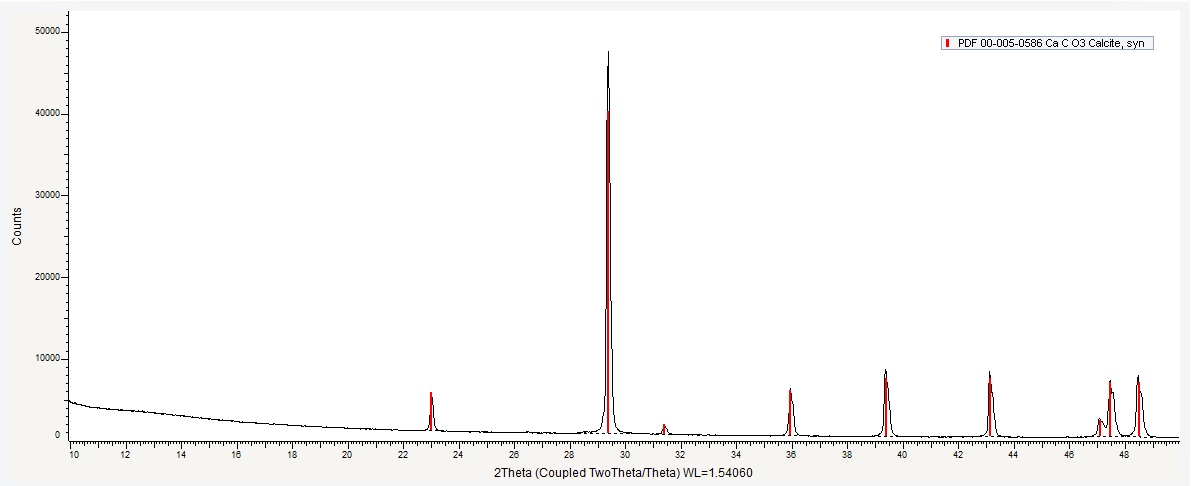

Supplement: S5 Data — (ZIP) [file pone.0188443.s007.zip › S6 Data/FURECULU.jpg]

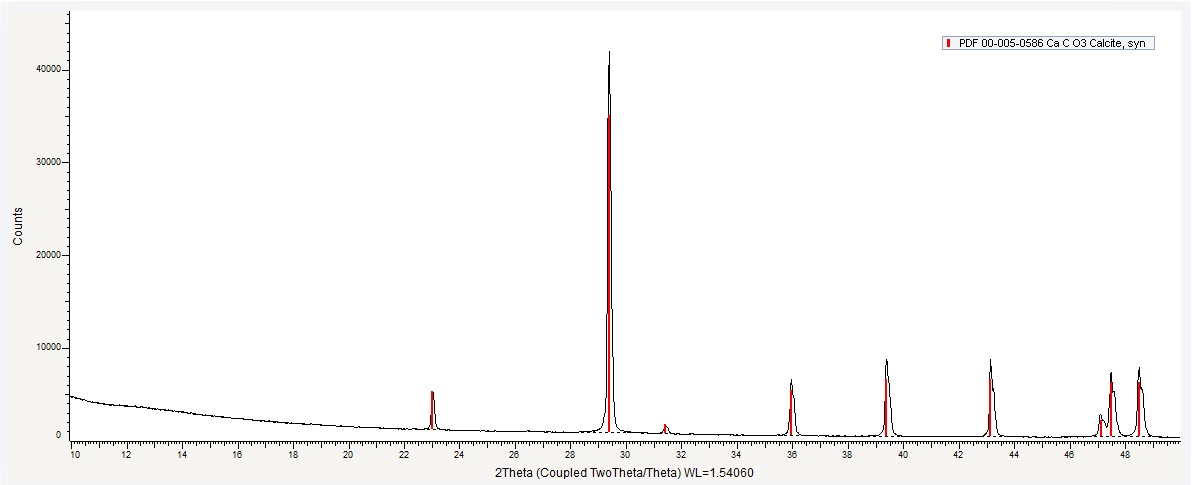

Supplement: S5 Data — (ZIP) [file pone.0188443.s007.zip › S6 Data/FUREMICO.jpg]

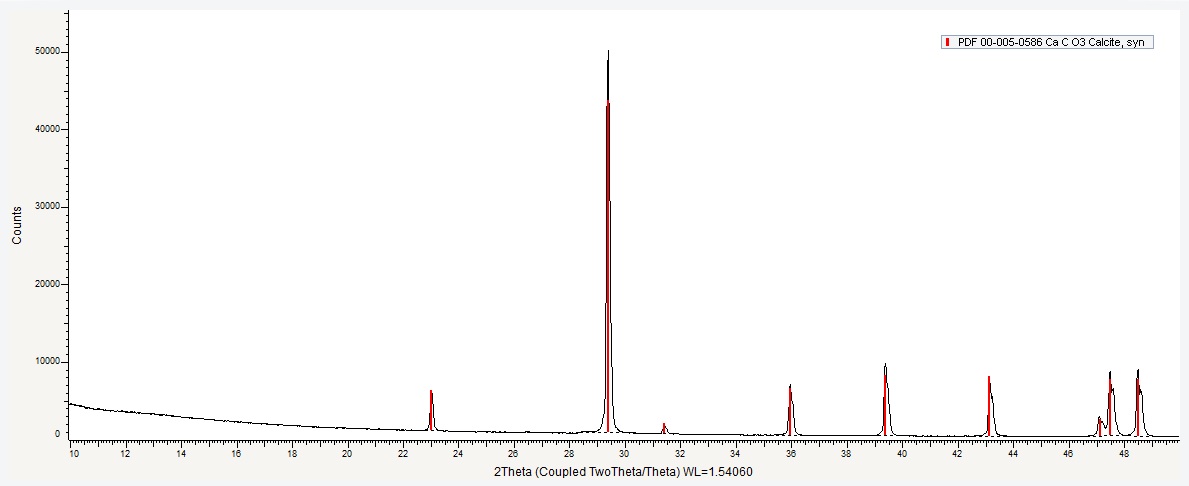

Supplement: S5 Data — (ZIP) [file pone.0188443.s007.zip › S6 Data/FUREMYRO.jpg]

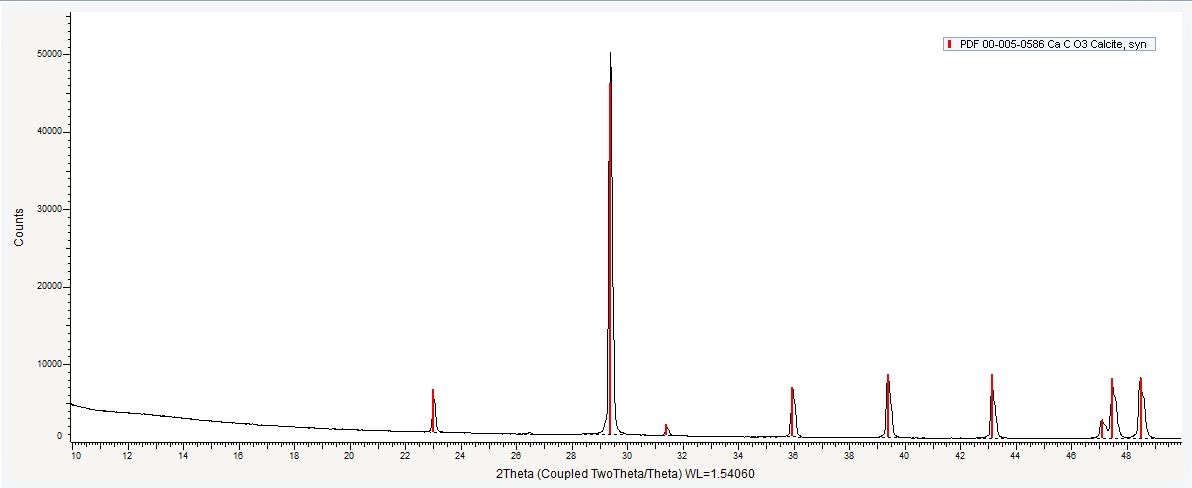

Supplement: S5 Data — (ZIP) [file pone.0188443.s007.zip › S6 Data/FUREPEMA.jpg]

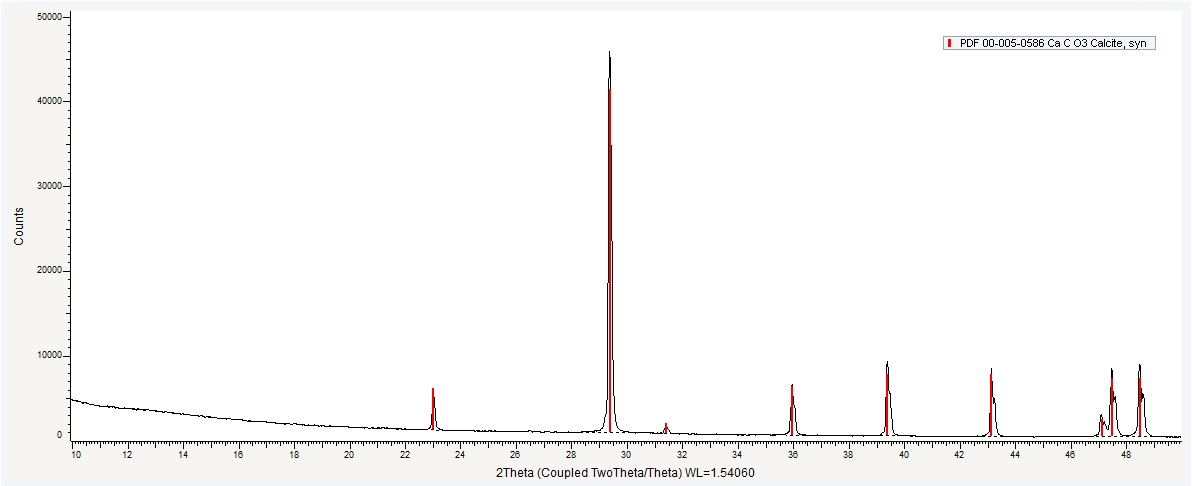

Supplement: S5 Data — (ZIP) [file pone.0188443.s007.zip › S6 Data/FUREPHEU.jpg]

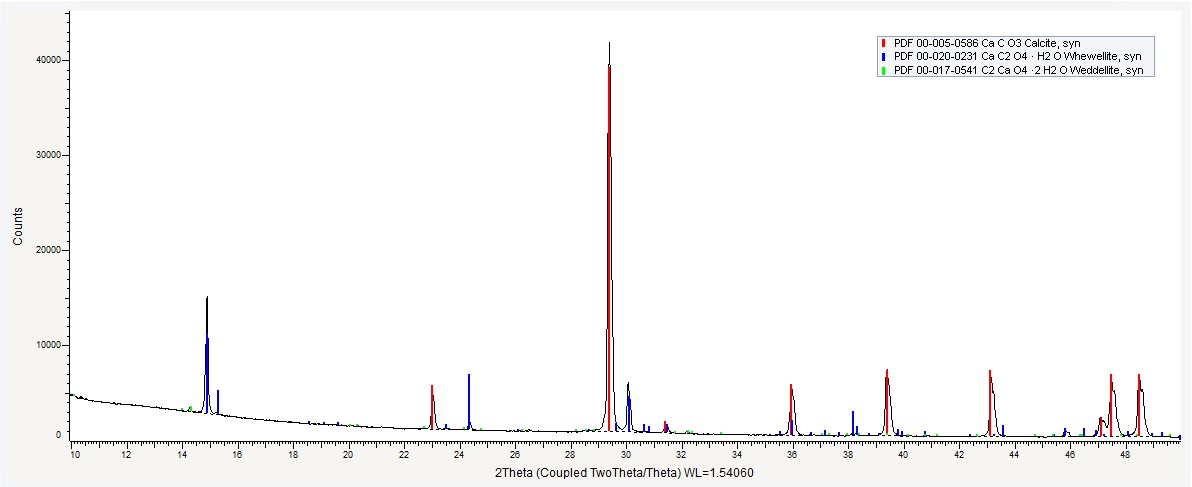

Supplement: S5 Data — (ZIP) [file pone.0188443.s007.zip › S6 Data/MICO.jpg]

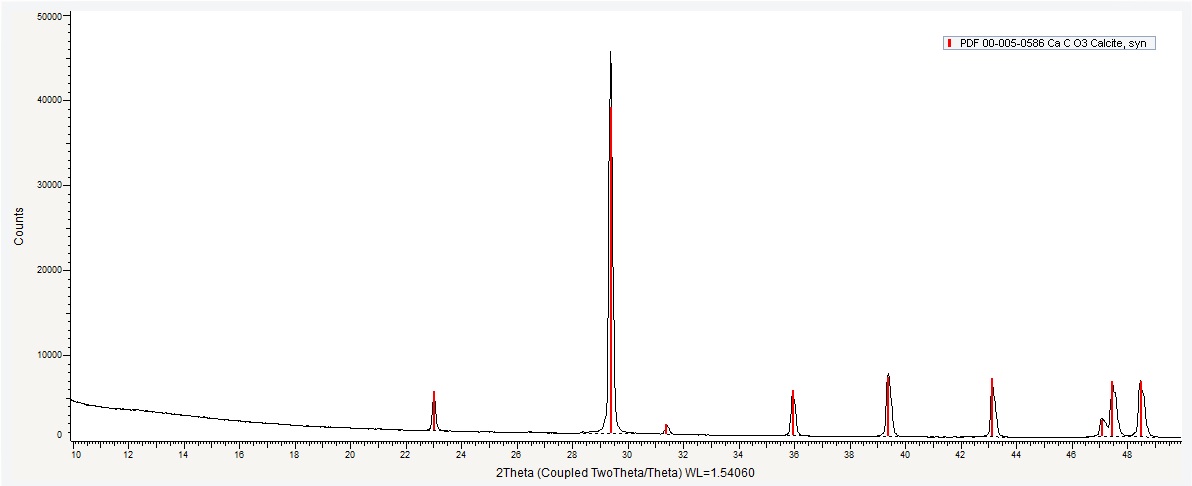

Supplement: S5 Data — (ZIP) [file pone.0188443.s007.zip › S6 Data/MICOMYRO.jpg]

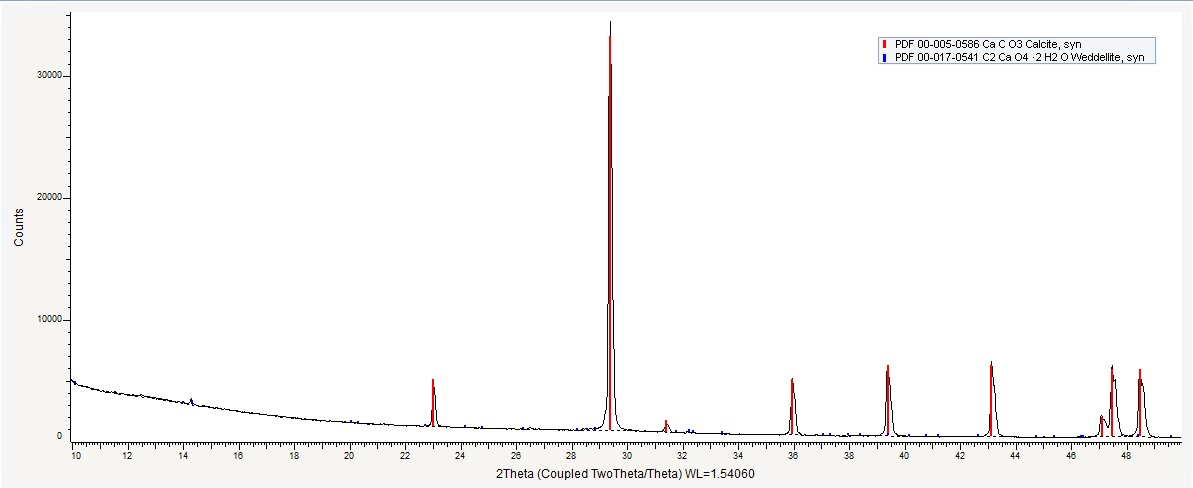

Supplement: S5 Data — (ZIP) [file pone.0188443.s007.zip › S6 Data/MICOPEMA.jpg]

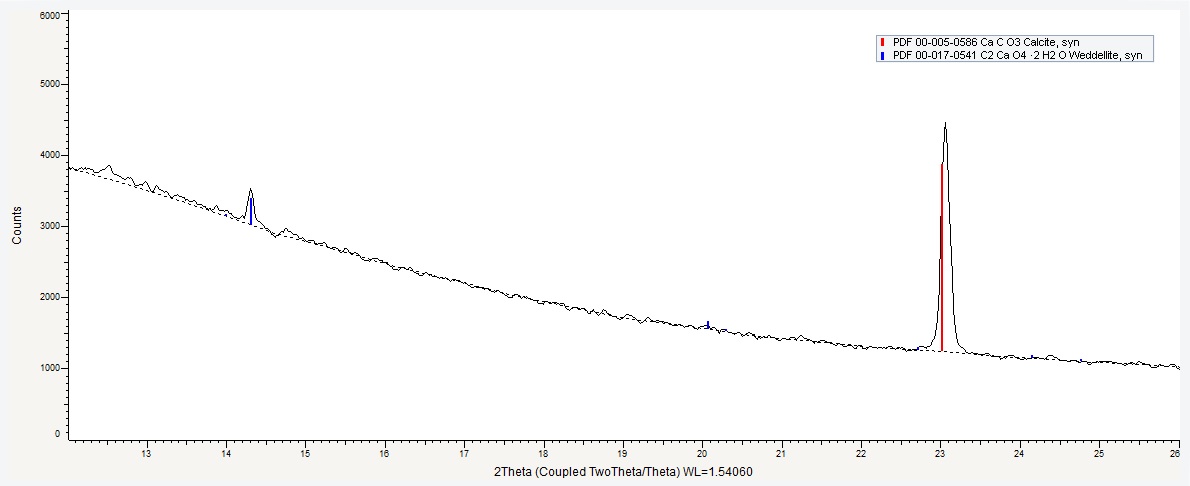

Supplement: S5 Data — (ZIP) [file pone.0188443.s007.zip › S6 Data/MICOPEMA_2.jpg]

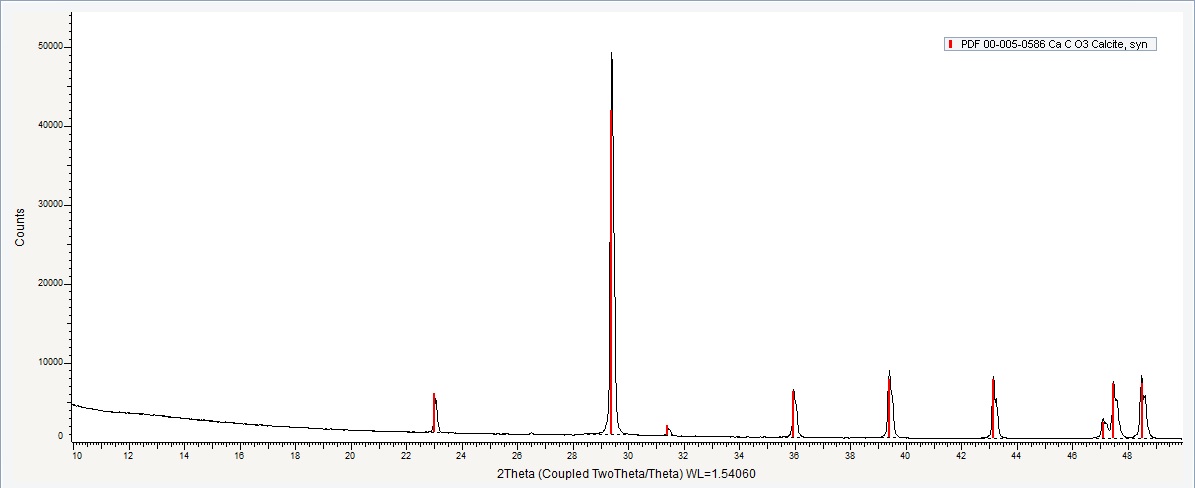

Supplement: S5 Data — (ZIP) [file pone.0188443.s007.zip › S6 Data/MYRO.jpg]

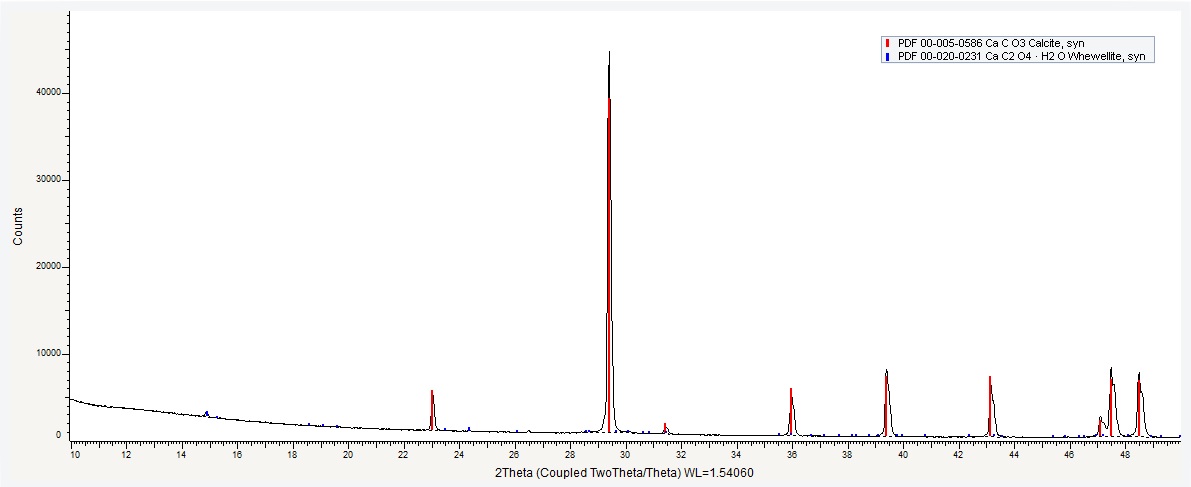

Supplement: S5 Data — (ZIP) [file pone.0188443.s007.zip › S6 Data/MYROPEMA.jpg]

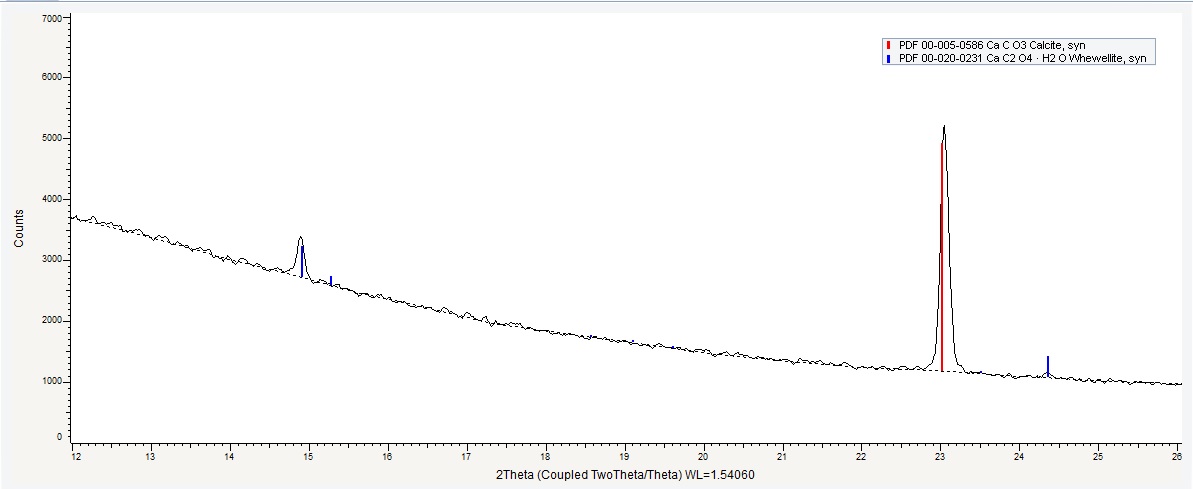

Supplement: S5 Data — (ZIP) [file pone.0188443.s007.zip › S6 Data/MYROPEMA_2.jpg]

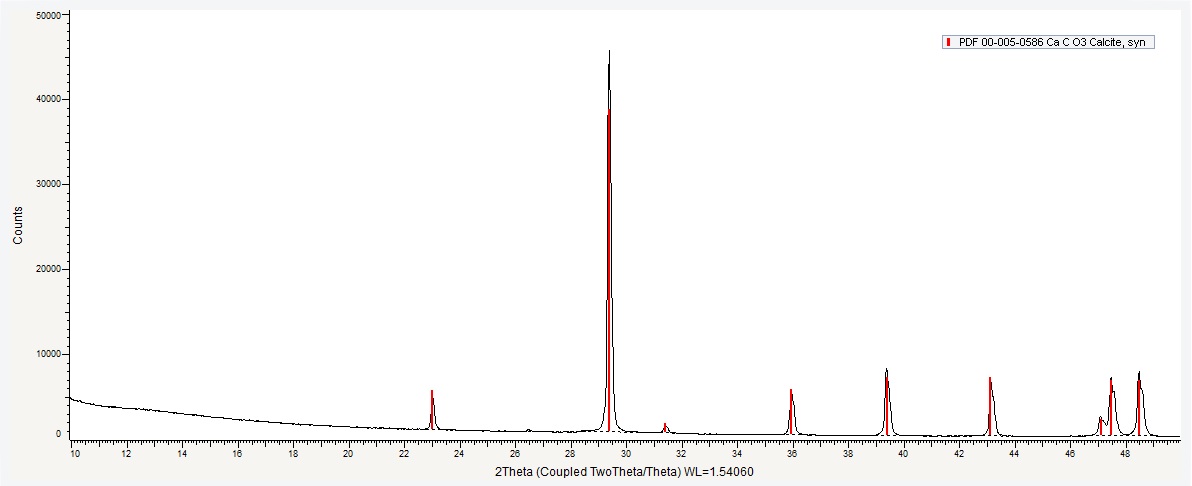

Supplement: S5 Data — (ZIP) [file pone.0188443.s007.zip › S6 Data/MYROPHEU.jpg]

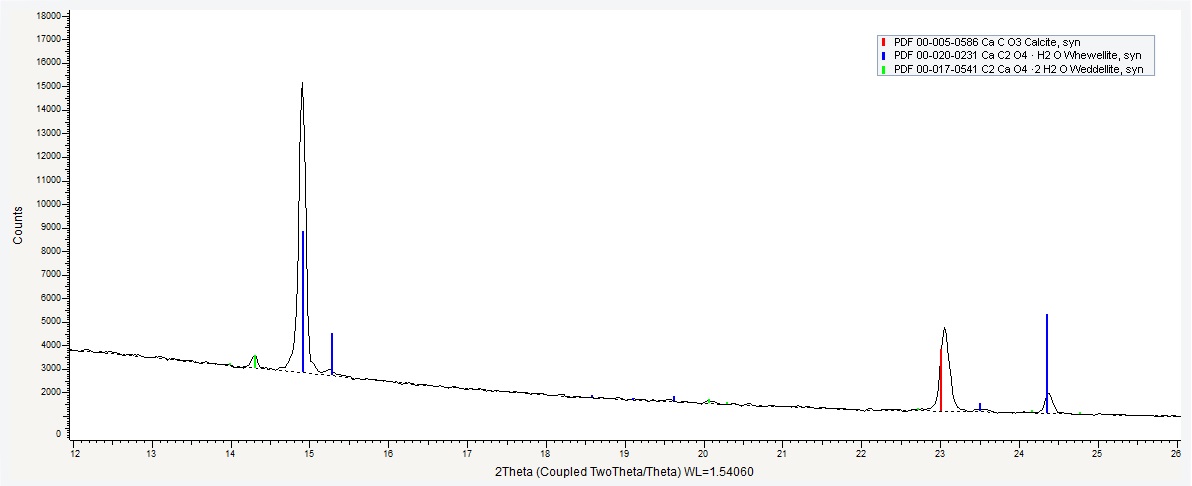

Supplement: S5 Data — (ZIP) [file pone.0188443.s007.zip › S6 Data/Paraconiothyrium sp.jpg]

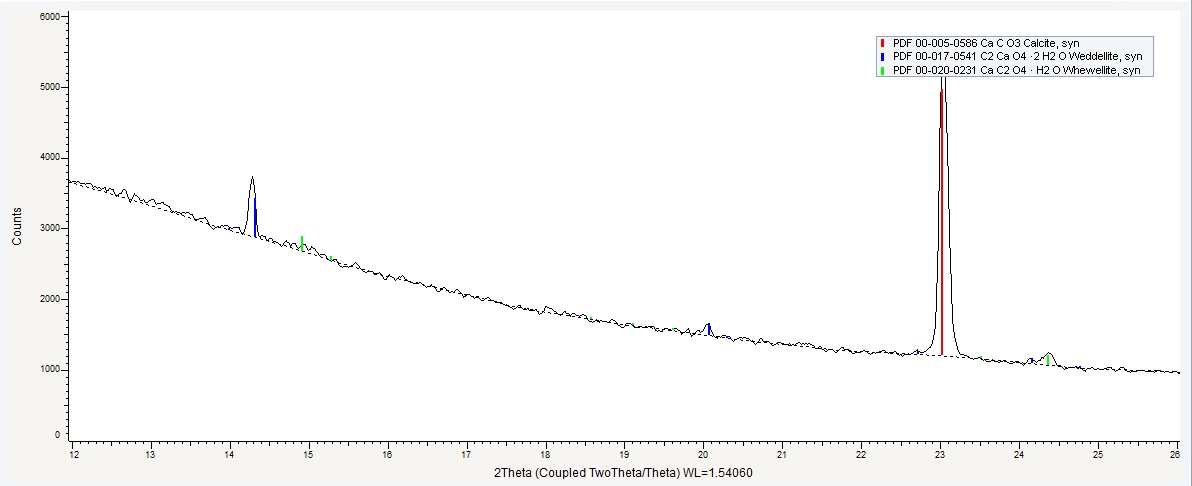

Supplement: S5 Data — (ZIP) [file pone.0188443.s007.zip › S6 Data/PEMA_2.jpg]

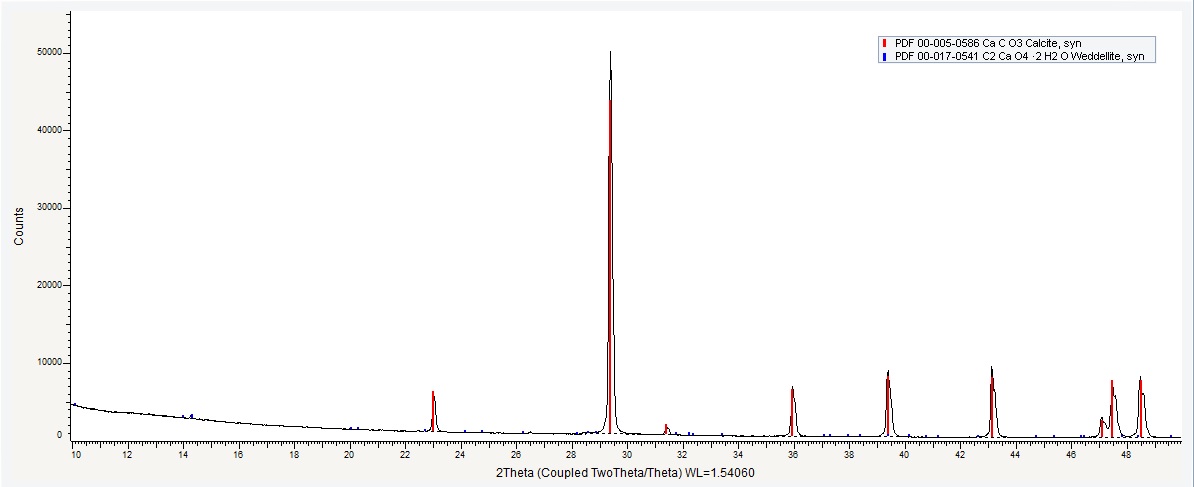

Supplement: S5 Data — (ZIP) [file pone.0188443.s007.zip › S6 Data/PEMAPHEU.jpg]

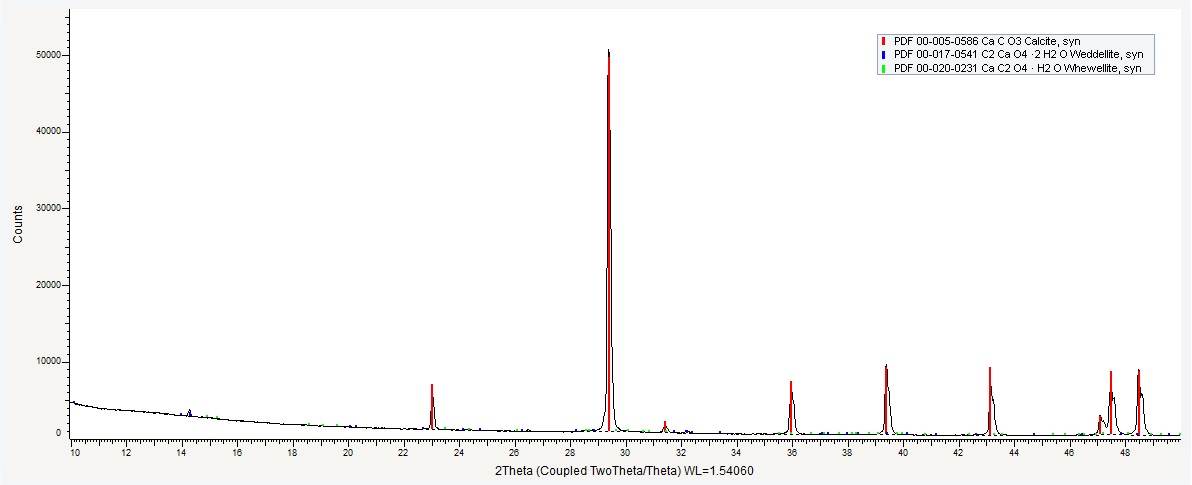

Supplement: S5 Data — (ZIP) [file pone.0188443.s007.zip › S6 Data/Pestalotiopsis maculans.jpg]

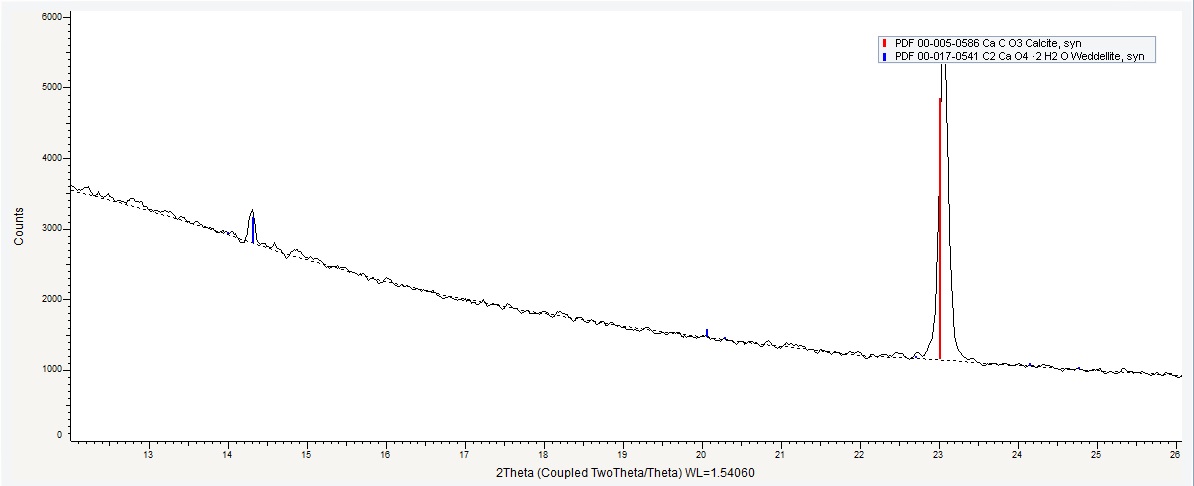

Supplement: S5 Data — (ZIP) [file pone.0188443.s007.zip › S6 Data/PHEMAPHEU_2.jpg]

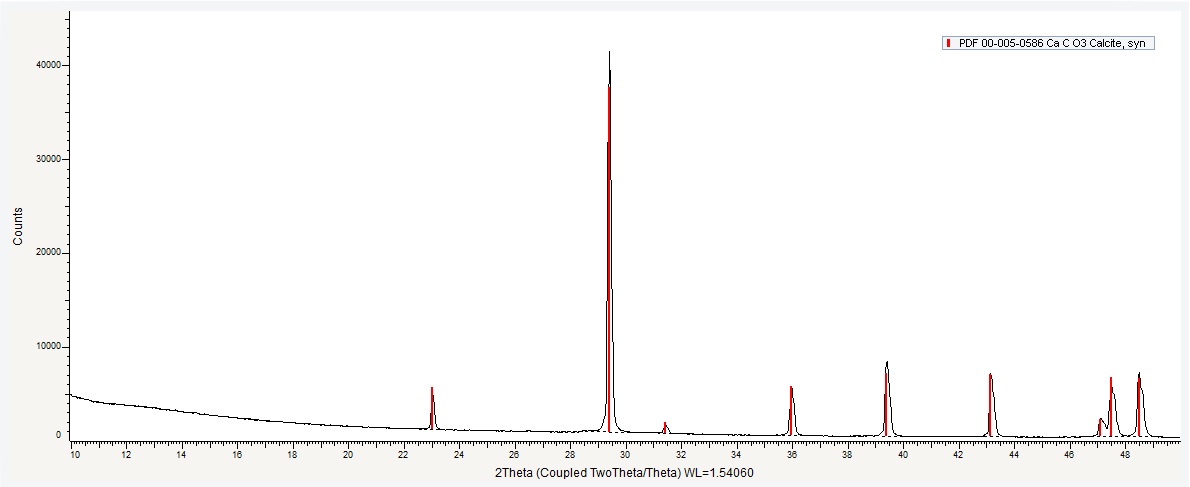

Supplement: S5 Data — (ZIP) [file pone.0188443.s007.zip › S6 Data/PHEU.jpg]

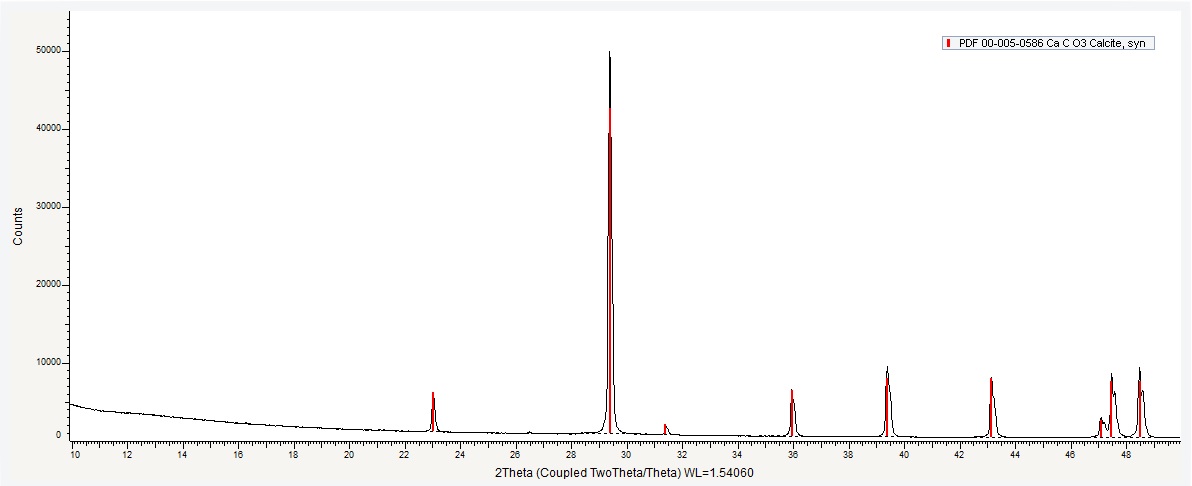

Supplement: S5 Data — (ZIP) [file pone.0188443.s007.zip › S6 Data/PHEUMICO.jpg]
